# Anger, agency, risk and action: a neurobehavioral model with proof-of-concept in healthy young adults

**DOI:** 10.3389/fpsyg.2023.1060877

**Published:** 2023-05-30

**Authors:** Tara L. White, Meghan A. Gonsalves, Chloe Zimmerman, Hannah Joyce, Ronald A. Cohen, Uraina S. Clark, Lawrence H. Sweet, Carl W. Lejuez, Adam Z. Nitenson

**Affiliations:** ^1^Department of Behavioral and Social Sciences, Center for Alcohol and Addiction Studies, and Carney Institute for Brain Science, Brown University, Providence, RI, United States; ^2^Neuroscience Graduate Program, Brown University, Providence, RI, United States; ^3^Undergraduate Program in Cognitive Neuroscience, Brown University, Providence, RI, United States; ^4^Center for Cognitive Aging and Memory, McKnight Brain Foundation, University of Florida, Gainesville, FL, United States; ^5^Department of Neurology, Icahn School of Medicine at Mount Sinai, New York, NY, United States; ^6^Department of Psychology, University of Georgia, Athens, GA, United States; ^7^Provost and Executive Vice President, Department of Psychology, Department of Psychiatry and Behavioral Health, Stony Brook University, Stony Brook, NY, United States

**Keywords:** incentive motivation, catecholamines, monoamines, reward, agency, anger, emotion

## Abstract

**Introduction:**

Anger can engender action by individuals and groups. It is thus important to understand anger’s behavioral phenotypes and their underlying neural substrates. Here, we introduce a construct we term *agentic anger*, a negatively valenced internal state that motivates action to achieve risky goals. We evaluate our neurobehavioral model via testable hypotheses in two proof-of-concept studies.

**Study 1 Methods:**

Study 1 used the Incentive Balloon Analogue Risk Task in a within-subjects, repeated measures design in 39 healthy volunteers to evaluate: (a) impact of blockade of reward on agentic anger, assessed by self-reports of negative activation (NA), (b) impact of achievement of reward on exuberance, assessed by self-reports of positive activation (PA), (c) the interrelationship of these valenced states, and (d) their relationship with personality.

**Study 1 Results:**

Task-induced NA was positively correlated with task-induced PA, risk-taking on the task and trait Social Potency (SP), a measure of trait agency and reward sensitivity on the Multidimensional Personality Questionnaire Brief-Form.

**Study 2 Methods:**

Study 2 assessed functional MRI response to stakes for risk-taking in healthy volunteers receiving 20 mg *d*-amphetamine in a double-blinded, placebo-controlled crossover design (*N* = 10 males), providing preliminary information on ventral striatal response to risky rewards during catecholamine activation.

**Study 2 Results:**

Trait SP and task-induced PA were strongly positively related to catecholamine-facilitated BOLD response in the right nucleus accumbens, a brain region where DA prediction error signal shapes action value and selection. Participants’ task-induced NA was strongly positively related with trait SP and task-induced PA, replicating the findings of Study 1.

**Discussion:**

Together these results inform the phenomenology and neurobiology of agentic anger, which recruits incentive motivational circuitry and motivates personal action in response to goals that entail risk (defined as exposure to uncertainty, obstacles, potential harm, loss and/or financial, emotional, bodily, or moral peril). Neural mechanisms of agency, anger, exuberance, and risk-taking are discussed, with implications for personal and group action, decision-making, social justice, and behavior change.

## Introduction

Anger encourages action by individuals and groups that can have positive or negative consequences. Violence and aggression motivated by anger are worldwide problems with major sociological and economic impact (Krug et al., 2002). However, anger can also motivate positive, prosocial action in response to social injustice (Brosnan and de Waal, 2014; Lindebaum and Geddes, 2016). Malcolm X noted “Usually when people are sad, they do not do anything…But when they get angry, they bring about a change” (Corrielus, 2021). Poet-activist Maya Angelou states “I believe in anger. Anger’s like fire, it can burn out all the dross and leave some positive things” (Sallis, 2019). There is thus potentially great value in understanding the triggers, phenomenology, and sequelae of anger relevant to goal-related targets and their supporting neurobiology.

Research definitions of anger have fluctuated, complicating progress in understanding this emotion. Early work defined anger largely in terms of its relationship with physiological arousal (Ax, 1953). Since then, anger has been variously conceptualized as (a) a basic emotion that is universally recognized (Ekman, 1992); (b) an emotional state that varies along a dimension of “feelings that vary in intensity, from mild irritation or annoyance to intense fury and rage” (Spielberger et al., 1983); (c) a multidimensional state shaped by cultural context, motivation, internal awareness, behavioral changes, physiologic arousal, and motor actions (Eckhardt and Deffenbacher, 1995; Kassinove and Sukhodolsky, 1995; Eckhardt et al., 2004); and (d) an affective state actively constructed by individuals, yielding idiosyncratic anger triggers, experience, expression and neural correlates (Touroutoglou et al., 2015). Operationalization of anger is further complicated by difficulty in distinguishing anger from other negatively valenced states, such as hostility, aggression, and rage, which can be fleeting or infrequent (Suarez and Williams, 1989; Fredrickson et al., 2000). Moreover most studies focus on the negative outcomes of anger, with less attention to positive processes such as ‘moral anger.’ This state is an emotional response to perceived injustice, unfairness, or norm violations, and can promote positive actions and interventions which would not otherwise be taken by the individual (Fischer and Roseman, 2007; Dijker, 2010; Brosnan and de Waal, 2014). To address these gaps, we take a neurobehavioral approach to anger, agency, and risk.

### A neurobehavioral model of agentic anger and valenced agentic emotion

We propose a subtype of anger – ‘*agentic anger*’ – that exists along a continuum of valenced emotion to motivate personal action in response to goals which entail risk. In this context, *risk* is defined as exposure to uncertainty, obstacles, potential harm, loss, and/or financial, emotional, bodily, or moral peril. Agency is a major domain of healthy functioning that provides the ability to shape one’s own choices and action in the world (White and Gonsalves, 2021). Traits of agency involve goal-directed behavior, incentive motivation and leadership, and facilitate positive emotional states with an activational component, such as vigor, engagement and enthusiasm (Depue and Collins, 1999; Grodin and White, 2015; White et al., 2021; White and Gonsalves, 2021). As such, agency is directly relevant to a number of anger-related processes, including action selection and approach of goal-related targets that pose personal risk. As risk exists along a continuum of intensity and varies in import – from the merely irritating to imminent mortal or existential threat – emotional response to these risks will vary in both intensity and duration. These responses will be modulated by a number of factors, including but not limited to one’s surrounding context, personal history, and physiological state. Given these factors, agentic anger and related states are likely to emerge along a continuum in healthy individuals. At the low end of activation, there will be an absence of elicitation of negatively valenced agentic states, producing a sense of calm; and at the high end of activation, there will be strong to very strong elicitation, producing intense emotion (for instance, extreme anger, outrage, and behavioral activation; representing very strong activation of activated negative emotion). Thus agentic anger will emerge along a continuum of intensity in healthy people, depending on the severity and duration of the eliciting trigger, the surrounding context, and one’s internal physiological state. This dimensional expression is also observed for positively valenced agentic states, such as exuberance, which emerge along a natural continuum of intensity, frequency and duration in healthy individuals (see Depue and Collins, 1999).

Several lines of evidence support such an agentic view of anger. While healthy adults often report that they experience anger as aversive and try to avoid becoming angry (Watson et al., 1999), anger also increases the frequency and intensity of events that are positive in tone, such as feeling proud, optimistic and wanting to take immediate action (Mackie et al., 2000; Lerner and Keltner, 2001; Harmon-Jones et al., 2004). Anger also has adaptive value. For instance, anger signals a dominant status in social settings (Tiedens, 2001), and is reported by elite athletes during competitive sporting events, where it facilitates optimal athletic performance (Robazza et al., 2006; Uphill and Jones, 2007; Maxwell and Visek, 2009; Woodman et al., 2009; Lane et al., 2012). Combat veterans report anger on the battlefield as a protective, positive force that facilitates their personal survival (Adler et al., 2008, 2017; Shea et al., 2018). For women in situations of domestic abuse, anger motivates decisions and ability to leave their abusers, a step typically accompanied by an increased risk of personal harm (Choi et al., 2009). In Korean culture this anger has a name – *Hwa-Byung* – and is particularly instrumental in women without other resources (Choi et al., 2009). In studies in the U.S., suppression of anger has been observed to mediate the relationship of women’s prior history of abuse as children and their later re-victimization by an intimate life partner (Maneta et al., 2012). Developmentally, positive associations of anger, joy, and goal-directed effort emerge in healthy infants as young as 2 months of age, indicating an early neurodevelopmental association of anger, positive emotion and reward processing (Lewis et al., 1990; Tamir and Ford, 2012).

The above literature suggests that anger involves incentive motivation, the internal drive to approach achievable rewards (Bindra, 1968). Incentive motivation is attuned to external rewards such as food, partners, status, and safety, and facilitates voluntary approach and acquisition of these rewards through the activation of meso-cortical and meso-limbic dopamine circuitry (Gray, 1982; Gray, 1987; Fowles, 1988; Depue and Collins, 1999; Harmon-Jones, 2003; Depue and White, 2015; Grodin and White, 2015). Agentic anger may thus affect the perception, processing, and approach of risky yet achievable rewards as part of a larger system of incentive motivation in humans.

Mechanistically, valenced agentic states such as anger and exuberance likely involve catecholaminergic (CA) activation in the ventral striatum in healthy individuals. This relationship is consistent with a growing evidence base on phasic dopamine (DA) reward utility prediction error signal (‘DA prediction error’) in the region, and dopaminergic theories of positive agentic states in healthy young adults (Depue and Collins, 1999). In healthy individuals, DA action in striatum involves both ambient (tonic) DA which relates to movement, and phasic DA which relates to reward (Schultz, 2016b). Prior to reward roughly a third of striatal neurons show phasic DA responses that vary as a function of reward risk [operationalized as a combination of variance and skewness (Fiorillo et al., 2003)], and 70–90% of striatal neurons show post-reward ‘DA prediction error’ responses (Schultz, 1998; Nomoto et al., 2010; Fiorillo et al., 2013). This latter DA prediction error signal has two components (Schultz, 2016b). The first component is an initial, unselective salience response, which is sensitive to the intensity and novelty of the eliciting stimulus (Schultz and Romo, 1990; Ljungberg et al., 1992; Fiorillo et al., 2013). The second component is a phasic DA reward response, which codes reward value as a prediction error (Schultz, 2016b). These DA prediction errors can be positive, negative, or bidirectional. Moreover, reward value is coded as subjective and varies across individuals, risk conditions, and personal risk attitudes (Sugam et al., 2012; Lak et al., 2014; Schultz, 2016b). DA prediction error signal thus encodes an individual’s subjective valuation of risk and reward at time of testing. This signal is believed to act in Hebbian fashion to strengthen synaptic efficacy of circuits and ensembles connecting specific behaviors with reward, with positive DA prediction values *strengthening* synaptic connections and negative DA prediction values *weakening* these same connections (Schultz, 1998; Schultz, 2015, 2016b). In this manner, positive DA prediction error signal actively shapes the responses of ‘action value neurons’, those VS cells that encode the reward value of specific actions (Sutton and Barto, 1981; Schultz, 2016b). Action values then provide an input to decision-making, guiding voluntary behavior according to the individual’s personal, subjective valuation of reward and risk in the moment (Schultz, 2016b).

Building on these findings, we here propose a neurobehavioral model with a specific profile of eliciting triggers, subjective phenomenology, and neural circuitry for valenced agentic states, such as exuberance and agentic anger in healthy humans (Table 1). Our model is inspired by (and consistent with) prior theoretical and empirical work on positively valenced agentic states, traits, and incentive phenomena in healthy persons (Depue and Collins, 1999).

**Table 1:** Neurobehavioral model of agentic anger and valenced agentic emotion.

	Levels of analysis				
Model tenets (T) and study hypotheses (H)	Stimuli	States	Traits	Behavior	Circuits
**Coherence of agentic stimuli, states, behavior, traits, and circuits**					
AGENTIC STIMULI: Risky rewards should induce agentic emotion of both positive and negative valence (exuberance, anger; jointly referred to as ‘valenced agentic emotion’) *H1-a. Blockade of worked-for risky rewards (‘blockade of reward’ or ‘blockade’) is an eliciting stimulus that triggers agentic anger and negatively valenced agentic states* *H1-b. Achievement of worked-for risky rewards (‘achieved reward’ or ‘achievement’) is an eliciting stimulus that triggers exuberance and positively valenced agentic states*	✓	✓			
AGENTIC STATES: Valenced agentic emotion (exuberance, anger) should correlate positively within- and between-persons *H2. Blockade-induced agentic anger will correlate with success-induced exuberance*	✓	✓	✓		
AGENTIC BEHAVIOR: Valenced agentic emotion (anger, exuberance) should motivate action and voluntary approach of risky rewards *H3-a. Blockade-induced agentic anger will motivate the volitional approach of risky rewards* *H3-b. Achievement-induced exuberance will motivate the volitional approach of risky rewards*	✓	✓		✓	
AGENTIC TRAITS: Valenced agentic emotion (anger, exuberance) should be shaped by traits that index the sensitivity to reward *H4-a. Blockade-induced agentic anger will relate to trait agency (trait social potency)* *H4-b. Achievement-induced exuberance will relate to trait agency (trait social potency)*	✓	✓	✓		
AGENTIC CIRCUITS: Catecholaminergic circuit reactivity should relate to the frequency, intensity and duration of valenced agentic emotion (anger, exuberance) in natural settings *H5-a. Stimulus-induced agentic anger will correlate with intrinsic reactivity of catecholamine circuitry (AMP-facilitated BOLD)* *H5-b. Stimulus-induced exuberance will correlate with intrinsic reactivity of catecholamine circuitry (AMP-facilitated BOLD)*	✓	✓	✓		✓
**Convergent validity**					
CONVERGENT STATES: Valenced agentic emotion (anger, exuberance) should be positively related to other agentic states in the same person (convergent validity) *H6-a. Blockade-induced agentic anger will correlate with other valenced agentic emotion (elation)* *H6-b. Achievement-induced exuberance will correlate with other valenced agentic emotion (elation)*	✓	✓	✓		
**Discriminant validity**					
DIVERGENT STATES: Valenced agentic emotion (anger, exuberance) should be independent of non-agentic states (discriminant validity) *H7-a. Blockade-induced agentic anger will be independent of non-agentic states (state anxiety, subjective arousal, physiological arousal)* *H7-b. Achievement-induced exuberance will be independent of non-agentic states (state anxiety, subjective arousal, physiological arousal)*	✓	✓	✓		
DIVERGENT TRAITS: Valenced agentic emotion (anger, exuberance) should be independent of non-agentic traits (discriminant validity) *H7-a. Blockade-induced agentic anger will be independent of non-agentic traits (trait impulsivity, aggression, anxiety, planfulness, affiliation, fear, immersive emotion)* *H7-b. Achievement-induced exuberance will be independent of non-agentic traits (trait impulsivity, aggression, anxiety, planfulness, affiliation, fear, immersive emotion)*	✓	✓	✓		
**Predictive validity**					
TEMPORAL PREDICTION: Frequency, intensity and duration of agentic emotion, risk-taking, and incentive reactions at one time period should provide information about their frequency, intensity and duration at other time periods (predictive validity) *H8-a. Stimulus-induced agentic anger at one time period will predict the frequency, intensity and duration of agentic anger at other time periods* *H8-b. Stimulus-induced exuberance at one time period will predict the frequency, intensity and duration of exuberance at other time periods*	✓	✓	✓	✓	✓

Aim: To evaluate contributions of agentic processes to anger and other negatively and positively valenced emotional states, with attention to limits on these contributions (i.e., ‘boundary conditions’). Neurobehavioral models of emotion involve differing levels of analysis, and include eliciting stimuli, affective states, affective traits, activated behavior, and supporting circuits (columns). Model tenets (T) and study hypotheses (H; italics) are in rows. Level of analysis in checkmark.

Our model is summarized in Table 1 and aims to evaluate contributions of agentic processes to anger and other valenced activated states, with attention to limits on these contributions (i.e., ‘boundary conditions’). This approach discriminates the extent to which agency shapes – or fails to shape – state and trait anger in specific contexts, and provides multiple levels of analysis, consistent with prior neurobehavioral models of incentive phenomena (e.g., Depue and Collins, 1999). Relevant levels of analysis include (1) stimuli eliciting the emotion, (2) activated states’ subjective phenomenology, (3) ensuing behavioral sequelae, (4) traits modulating these responses, and (5) neurocircuitry supporting these responses (‘stimuli’, ‘states’, ‘traits’, ‘behavior,’ and ‘circuits’ in Table 1).

An agentic approach to anger predicts a specific constellation of observable phenomena in healthy individuals. These tenets, summarized in Table 1, are described below, and can be evaluated in specific directional hypotheses in a wide variety of natural and experimental settings.

First, if anger, exuberance and other valenced agentic states are elicited by risky rewards important to the individual, there should be an observable relationship between these stimuli and states (model tenet 1 (T1), Table 1). We evaluate this in hypothesis 1: that blockade of worked-for risky rewards (‘blockade of reward’) should trigger anger and other activated negative emotion; and acquisition of those same rewards (‘achieved reward’) should trigger exuberance and activated positive emotion, such as enthusiasm, joy, engagement and vigor (Depue and Collins, 1999; Grodin and White, 2015) (hypothesis H1, Table 1).

Second, if anger, exuberance and other valenced activated states entail an agentic component, such states should be positively associated, due to contributions of incentive motivational processes to each outcome (model tenet 2, Table 1). This prompts hypothesis 2: that stimulus-induced agentic anger and stimulus-induced exuberance should positively associate within- and across-persons. This relationship should be particularly evident during active risk-taking, when reward-related cues, intermittent achievement of reward, and intermittent blockade of reward are interspersed, thereby providing direct, interleaved challenge of both anger and exuberance. We thus expect internal subjective experiences of positively and negatively valenced agentic emotion –here, exuberance and anger – to be positively correlated, such that individuals who are ‘fast to anger’ are ‘fast to joy’, while those who are ‘slow to anger’ are also ‘slow to joy’ (hypothesis H2, Table 1).

Third, if anger entails an agentic component, this and other valenced agentic states should motivate the voluntary approach of risky rewards, goals, and targets (model tenet 3, Table 1). This prompts hypothesis 3: that stimulus-induced anger and other activated negative emotion should facilitate the volitional approach of risky rewards in the environment; and stimulus-induced exuberance (and related states) should similarly facilitate volitional approach of these rewards (hypothesis H3, Table 1).

Fourth, if anger, exuberance and other valenced states entail an agentic component, these states should collectively relate to traits that index the intrinsic reactivity to reward, thereby modulating both *negatively* and *positively* valenced reactions to external events (model tenet 4, Table 1). This prompts hypothesis 4: that trait-levels of agency (‘trait agency’) should predict the frequency, intensity and magnitude of negatively valenced agentic emotion when worked-for rewards are withheld (e.g., low to high-intensity states of irritation, annoyance, tension, frustration, and anger), as well as the frequency, intensity and magnitude of positively valenced agentic emotion when worked-for rewards are achieved (e.g., low to high-intensity states of positive engagement, excitement, vigor, exuberance and surgency; hypothesis H4, Table 1).

Fifth, if anger, exuberance and other valenced states entail an agentic component, then the intrinsic reactivity of incentive motivational circuits and networks – including ventral striatum (VS), involved in action selection – should shape the frequency, intensity and magnitude of both agentic anger and exuberance experience (model tenet 5). This prompts hypothesis 5: that stimulus-induced anger and related negative states should correlate with activation of catecholamine circuitry in healthy individuals, and stimulus-induced exuberance and related positive states should similarly correlate with this activation (hypothesis H5, Table 1).

Last, we propose three boundary conditions: that agentic anger and other valenced agentic emotion should display convergent, discriminant, and predictive validity. Regarding convergent validity (model tenet 6), anger and exuberance should correlate with other agentic states reported by individual. This prompts hypothesis 6: that stimulus-induced agentic anger will correlate with other valenced agentic states (e.g., elation), and stimulus-induced exuberance will also correlate with these states (hypothesis H6, Table 1).

Regarding discriminant validity (model tenets 7–8), if anger and exuberance entail an agentic component, then these states should be independent of states without an agentic component. This is tested in hypothesis 7: that stimulus-induced agentic anger and exuberance are independent of non-agentic states (such as anxiety and general arousal; H7, Table 1). Similarly, personality traits without an agentic component – such as trait anxiety, affiliation, immersive emotion, fear/cautious timidity, impulsivity/planfulness, and interpersonal aggression – should be largely unrelated to the frequency, intensity, and duration of anger and exuberance evoked by risky reward (tenet 8). This is tested in hypothesis 8: that stimulus-induced anger should be independent of non-agentic traits; and stimulus-induced exuberance should be similarly independent (hypothesis H8, Table 1).

For temporal and predictive validity (tenet 9), we expect agentic states, traits, behaviors, and circuits to interrelate in a predictable manner over time, with high test–retest and predictive validity (model tenet 9). This prompts hypothesis 9: that stimulus-induced agentic anger and exuberance at one time will predict the frequency, intensity and duration of agentic anger and exuberance at other time periods (hypothesis H9, Table 1).

This neurobehavioral model of agentic anger, articulated above and in Table 1, is consistent with known contributions of DA prediction error and pre-reward risk signals in VS, and can be evaluated in healthy individuals using a variety of primary and secondary reinforcers, such as monetary rewards, food rewards, pleasurable drug rewards, social cues, and emotion induction.

### Evaluation of the neurobehavioral model in healthy young adults

We evaluate the nine tenets of the agentic model in two proof-of-concept studies. Study 1 evaluates major tenets of the model (tenets T1–T4, T6–T9) in a laboratory setting, providing preliminary proof-of-concept data on agentic states, traits, and behavior in healthy young adults. Study 2 evaluates neural correlates and predictive validity (tenets T5, T9) in a functional MRI (fMRI) and *d*-amphetamine (AMP) drug challenge study and provides preliminary proof-of-concept data on the relationship of agentic states, traits, and VS response to risky rewards during activation of central and peripheral catecholamine (CA) circuitry. Both studies are conducted in healthy volunteers. Together the studies provide novel proof-of-concept preliminary data on anger as a valenced, dimensional agentic response, complementing and extending standard models of risky decision-making and DA prediction error (e.g., dual process models; prospect theory; fuzzy trace theory; behavioral economic), neurobiological and memory models; see (Kahneman and Tversky, 1979; Leahey, 2003; Birnbaum, 2008; Reyna and Rivers, 2008; Reyna et al., 2011; Rutledge et al., 2015; Schultz, 2016b; Diederich and Trueblood, 2018; Edelson and Reyna, 2021).

## Experiment 1

### Rationale and methods

Study 1 presented the incentive Balloon Analogue Risk Task (i-BART) to healthy young adult volunteers under standard laboratory conditions on two occasions in a repeated-measures, within-subjects design. The i-BART is an economic decision-making task that is relevant to processing of risk and reward and does not entail a prolonged learning component [e.g., behavior on the first 10 balloons is correlated with and a proxy for behavior on subsequent balloons; see (Lejuez et al., 2002, 2003)]. The task provides information on individual differences, state-related changes, and dimensional structure and phenomenology of risk behavior at the time of testing. As a behavioral economic task the iBART is *prima facie* relevant to reactions to systems that inflict financial unfairness and uncontingent nonreward. The iBART was selected as an initial test of agentic anger, providing information on affective responses relevant to tenets T1–T4 and T6–T9 in study 1 (Table 1).

We report how we determined our sample size, all data exclusions, all manipulations, and all measures in the study, and we follow Journal Article Reporting Standards (JARS) (Kazak, 2018). All data, analysis code, and research materials are available upon request. Data were analyzed using SPSS, version 25 (IBM, v.25).

### Participants

Thirty-nine healthy young adults were recruited from the University of Chicago and surrounding community and provided written informed consent. Inclusion criteria were chronological age 18–35 years, minimum high school education, fluency in English, being within 20% of ideal body weight and confirmed drug-free via urine screening on the study days. Minimum subject age was set at 18, the age at which personality can be stably measured by adult personality questionnaires such as the MPQ Brief Form. Age was truncated at 35, as the dopamine transporters that mediate the acute effects of d-amphetamine and other stimulants (e.g., cocaine) show a significant negative correlation with age (van Dyck et al., 2002). Exclusion criteria included serious medical conditions (history of cardiac, pulmonary, or liver problems), hypertension, abnormal EKG, current or past major psychiatric disorder including substance use disorder, prescription medication in the past 6 months (excluding antibiotics), a history of stroke, brain tumor, or seizure disorder, current unstable residence or working a night-shift, a history of adverse reactions to stimulant drugs, and current or planned pregnancy or lactation in women. Exclusions were assessed by in-person interview, medical intake, and medical exam. Psychiatric history was assessed using the M.I.N.I psychiatric interview and was verified in the intake portion of the medical exam by an independent medical professional. All participants provided written informed consent. The research was approved by the Institutional Review Board at The University of Chicago (White et al., 2007, 2008).

### Experimental design

Agentic reactions to the i-BART task were evaluated on the orientation (OR) session (‘day 1’) and the placebo (PBO) day conducted approximately 2 weeks apart in each participant [11.5 ± 9.1 d (White et al., 2007)]. These assessments were conducted within a larger study of drug effects (White et al., 2007). Drug effects on emotion and behavior have already been published (White et al., 2007). Risk behavior is published and demonstrates good test–retest stability across the OR and PBO days (White et al., 2008). The present data provide information on emotional responses to the task on the OR and PBO days and have not been previously reported.

### Incentive balloon analogue risk task (i-BART)

The i-BART was presented via computer (White et al., 2007, 2008). Conceptually, the task involves ‘pumping up’ balloons for money. Virtual balloons on the task can and do ‘explode,’ at which point participants lose the money they had accrued on that balloon. The explosion point of each balloon is unknown to participants (for discussion, see Lejuez et al., 2003). Procedurally, during each trial participants had the opportunity to approach reward by pressing a button to pump up a series of virtual on-screen balloon images, until either (a) they chose to cash out and collect the money they accrued on that trial; or (b) the trial reached its pre-determined explosion point, the balloon image exploded, an explosion sound was played, earnings on the trial were automatically forfeited, and the next trial would begin. The i-BART provided three levels of incentive stakes, presented in sixty intermixed trials of low stakes (LS, 20 trials), medium stakes (MS, 20 trials) and high stakes (HS, 20 trials) trials of 0.5 cents, 1.0 cents, and 5.0 cents per pump, respectively (White et al., 2007, 2008). The 60 balloon trials had an average explosion point of 64 pumps, with a range of 1 to 128 pumps (Lejuez et al., 2002; White et al., 2007, 2008). Approach of risky reward was assessed by the average number of finger presses on trials that were cashed-out, with a higher number of presses indicating stronger approach behavior [adjusted average pumps, the standard measure of risk-taking on the task (Lejuez et al., 2002; White et al., 2007, 2008); additional details in Supplementary Methods]. After completion of the 60th trial, participants were presented with a congratulations screen, an applause sound was played, and participants were informed of the total amount of money they earned on the task. Participants were debriefed, paid their monetary earnings from the task and compensated for their overall study participation at the study exit, conducted on a separate day at completion of the study (White et al., 2007, 2008). The task is well-studied in laboratory and field settings, relates to real-world risk-taking and has good test–retest reliability (Lejuez et al., 2002, 2003; White et al., 2007, 2008).

### Study measures and tests of hypotheses

#### State measures of agency

Positive Activation and Negative Activation Rating Scales (PARS, NARS; Morrone et al., 2000; Weyandt et al., 2018) were used to evaluate valenced agentic responses to the task. These measures provide a test of hypotheses 1 and 2 (relevant to model tenets T1 and T2), outlined at the top of Table 1. The 10 point rating scale of PARS positive activation (PA) assessed the relative absence to the strong presence of positively valenced agentic emotion: 1 = depressed/sluggish, 2 = dull/tired, 3 = pleasant/fresh, 4 = cheerful/lively, 5 = delighted/energetic, 6 = enthused/peppy, 7 = thrilled/strong, 8 = exuberant/vigorous, 9 = elated/exhilarated, 10 = ecstatic/invincible (Morrone et al., 2000). The separate 10-point rating scale of NARS negative activation (NA) assessed the relative absence to the strong presence of negatively valenced agentic emotion: 1 = placid, 2 = calm, 3 = relaxed, 4 = annoyed, 5 = tense, 6 = nervous, 7 = distressed, 8 = jittery, 9 = hostile, 10 = contemptful (Morrone et al., 2000; Weyandt et al., 2018). *Rationale*. The PARS and NARS measures provide rapid assessment of valenced agentic emotion before and after the i-BART task. Ratings on PARS PA correlate with participant ratings on the PANAS (Watson et al., 1988) when rated at the same time over 3 days [*r* = + 0.88, positive scale; *r* = + 0.89, negative scale (Morrone-Strupinsky, 2002)]. PARS PA discriminates stimuli with neutral, moderate or high incentive salience (Morrone et al., 2000), and increases in response to agentic pictures, methylphenidate and Adderall (Depue, 2006; Morrone-Strupinsky and Lane, 2007; Depue and Fu, 2013; Weyandt et al., 2018). NARS NA provides a general assessment of negatively valenced activated (agentic) emotion along a dimension of intensity, from the absence of negatively valenced activated emotion (e.g., placid, calm) to the moderate elicitation of negatively valenced activated emotion (e.g., annoyed, irritated, tense) to the strong to very strong elicitation of negatively valenced activated emotion (e.g., jittery, hostile, contemptful). This method reduces social desirability bias in NA responding due to participants’ *a-priori* attitudes toward anger reactivity, experience, and expression, while providing information on internal subjective states along a continuum of intensity relevant to real-world anger. *Timing*. Scales were administered immediately prior to and immediately following the i-BART task. Scales before the task instructed participants to rate their emotion “right now.” Scales after the task instructed participants to rate PA to cash-out events, and NA to explosion events. Task-induced PA thus represents a dimension of positive agentic emotion (exuberance) to achieved rewards (i.e., cashed-out trials), and task-induced NA represents a dimension of negative agentic emotion (providing information relevant to the dimension of agentic anger) to the blockade of worked-for risky rewards (i.e., exploded trials).

*Tests for Hypothesis 1*. Task effects on valenced agentic emotion were evaluated using a 2 × 2 repeated measures within-subjects ANOVA, with two levels of time (pre-task, post-task; evaluating task effects) and two levels of day (day 1, day 2; evaluating day effects). Follow-up paired samples *t*-tests were conducted to determine the nature of task and day effects on PA and NA.

*Tests for Hypothesis 2.* Summary scores of induced PA were calculated as post-task PA minus pre-task PA, providing information on the magnitude of task-induced exuberance. Summary scores of induced NA were similarly calculated as post-task NA minus pre-task NA, providing information on the magnitude of task-induced agentic anger in each individual. Relationship of induced PA and induced NA was evaluated by bivariate Pearson correlation to evaluate linear relationships between these continuous variables.

#### Behavioral measures of approach of risky rewards

Voluntary approach of risky rewards was assessed by the average number of finger presses on trials that were cashed-out, with a higher number of presses indicating stronger approach behavior [adjusted average pumps, the standard measure of risk-taking on the task (Lejuez et al., 2002; White et al., 2007, 2008); additional details in Supplementary Methods]. This measure provides primary test of hypothesis 3 (relevant to model tenet T3, Table 1). Two additional measures – money earned and number of balloons exploded – provide contextual information about participants’ engagement with the task and were evaluated in a secondary analysis.

*Tests for Hypothesis 3. Primary test.* Relationship of induced PA and induced NA with risk-taking on the task was evaluated by bivariate Pearson correlations to evaluate linear relationships. *Secondary tests*. Relationship of induced PA and induced NA with money earned and explosions were also evaluated, to provide information on contextual correlates.

#### Trait measure of agency

Personality traits were assessed using the Multidimensional Personality Questionnaire Brief Form (MPQ-BF, Patrick et al., 2002a), an empirically-derived instrument with an orthogonal factor structure. The primary trait of interest was social potency (SP), a measure of trait agency. This measure is relevant to incentive motivation and the sensitivity to reward in healthy individuals (White and Depue, 1999; Depue and White, 2015; Grodin and White, 2015), providing a test of hypothesis 4, relevant to tenet T4 (Table 1).

*Tests for Hypothesis 4.* Relationship of induced PA and induced NA with trait agency was evaluated by bivariate Pearson correlations.

#### Measures of construct and convergent validity

Subjective elation was evaluated 10 min prior to and 5 min following the task on day 2, providing data on validity. This measure provides a test of hypothesis 6 (relevant to model tenet T6, Table 1). Subjective elation was rated on a 100 mm visual analogue scale (VAS, Wewers and Lowe, 1990) ranging from ‘not at all’ to ‘extremely,’ providing a subjective measure of positive incentive tone.

*Tests for Hypothesis 6*.^1^ VAS Elation was evaluated for task effects (pre- vs. post-task) using paired samples *t*-tests. Summary scores were calculated as the difference between post- and pre-task VAS Elation, and relationship with induced PA and induced NA were assessed by Pearson correlation to provide information on construct and convergent validity.

#### Measures of specificity and discriminant validity

*State Measures*. Six measures of non-agentic states – subjective arousal, anxiety, and physiological arousal – were evaluated 10 min prior to and 5 min following the task on day 2, providing a test of Hypothesis 7 (relevant to model tenet T7, Table 1). The Profile of Mood States (POMS, McNair and Droppleman, 1971) Arousal scale provided a measure of subjective arousal. Physiological arousal was evaluated by systolic blood pressure, diastolic blood pressure, and heart rate. State anxiety was evaluated by the POMS anxiety scale (McNair and Droppleman, 1971) and a 100 mm VAS scale (Wewers and Lowe, 1990) with anxiety rated from ‘not at all’ to ‘extremely’.

*Tests for Hypothesis 7*. POMS arousal, POMS anxiety, VAS anxiety, systolic blood pressure, diastolic blood pressure, and heart rate before and after the task on day 2 were assessed for task effects using paired samples *t*-tests (pre- vs. post-task). Summary scores were calculated as the difference between post- and pre-task scores for each measure and entered into correlation analysis with induced PA and induced NA, providing information on state specificity and divergent validity.

*Trait Measures*. Six non-agentic traits were assessed to provide information on specificity and discriminant validity, providing evaluation of Hypothesis 8 (relevant to model tenet T8, Table 1). Non-agentic traits were (1) stress reaction (SR), a measure of trait anxiety and sensitivity to uncertainty and negative evaluation, relevant to sensitivity to negative feedback; (2) control (CON), a measure of trait behavioral planfulness as opposed to spontaneity, relevant to impulsivity; (3) social closeness (SC), a measure of trait affiliation rather than agency; (4) harm avoidance (HA), a measure of trait fear rather than anxiety; (5) absorption (ABS), a measure of mental–emotional flexibility and capacity for immersive emotion rather than behavioral flexibility; and (6) aggression (AG), a measure of trait hostility and intent to harm (White and Depue, 1999; Morrone et al., 2000; Patrick et al., 2002a; White et al., 2006a; Depue and White, 2010; Childs et al., 2014; Grodin and White, 2015; White et al., 2021).

*Tests for Hypothesis 8*. Relationship of induced PA and induced NA with non-agentic traits were evaluated by bivariate Pearson correlations to evaluate linear relationships, providing information on trait specificity and divergent validity.

#### Measures of predictive validity

State measures of agency (PA, NA) on the two study days, evaluated in the ANOVA (above), provided information on the stability and reproducibility of induced PA and NA over time. These inform hypothesis 9 (relevant to model tenet T9, Table 1).

*Tests for Hypothesis 9*. Change in task-induced agentic state from day 1 to day 2 was calculated as task-induced PA on day 2 minus task-induced PA on day 1, and task-induced NA on day 2 minus task-induced NA on day 1, providing information on change in task-induced agentic emotion over time.

#### Data quality and overall analysis

All participants had valid data on the MPQ-BF, PA, and NA (*N* = 39). All measures were evaluated for outliers (visually; Cook’s distance values >3 SD); there were no exclusions. One participant had missing data on VAS and POMS validity measures on day 2, reducing the sample size to *N* = 38 for these analyses.

Alpha was set at 0.05 for all tests. Directional hypotheses were evaluated using one-tailed thresholds and non-directional hypotheses were evaluated using two-tailed thresholds (per Table 1). Correlation coefficients were normalized using Fisher r to z transformation and compared to assess magnitude of relationships (Steiger, 1980). Effect sizes were calculated using methods of Friedman and Cohen (Friedman, 1968; Cohen, 1988). The study hypotheses were independent, and the majority were evaluated using single statistical tests (i.e., hypotheses 1, 2, 4, 6, and 9). Hypotheses involving multiple tests (i.e., hypotheses 3, 7, and 8) were corrected for multiple comparisons using the Bonferroni method. This method provided an adjusted alpha for criterion testing of 0.025 for two tests for hypothesis 3, 0.008 for six tests for hypothesis 7, and 0.008 for six tests for hypothesis 8, providing a conservative correction for multiple comparisons in these analyses.

### Results

#### Hypothesis 1. Task-induced exuberance and anger

**Figure 1:**
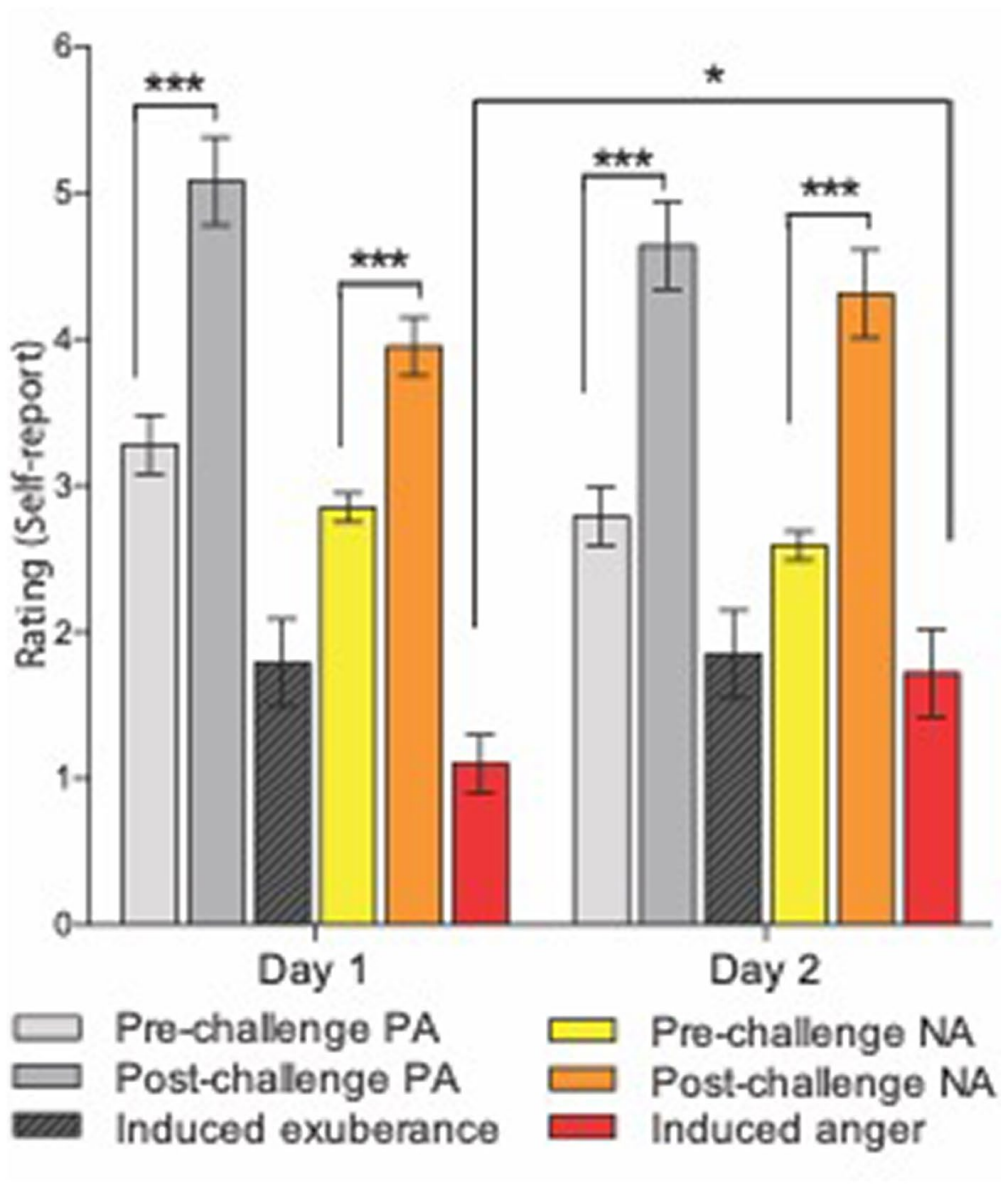
Experimental induction of agentic states. Means and standard errors for subjective response to the i-BART risk task on days 1 (left) and 2 (right). Challenge = Incentive Balloon Analogue Risk Task (i-BART). PA = Positive Activation (exuberance). NA = Negative Activation (agentic anger). Pre-task PA in light gray, post-task PA in dark gray, induced exuberance in black (post-task PA minus pre-task PA). Pre-task NA in yellow, post-task NA in orange, induced anger in red (post-task NA minus pre-task NA). The i-BART significantly increased anger (Negative Activation, NA) and exuberance (Positive Activation, PA) on both study days (*p* < 0.001). Rise in induced exuberance did not differ by day (n.s.). Rise in induced anger was larger on day 2 than day 1, evidence of behavioral sensitization (*p* < 0.001). Note, bars for Induced exuberance and Induced anger represent within-subject difference scores, calculated based on levels of a factor evaluated in the ANOVA (see methods). *p < 0.05, **p < 0.01, ***p < 0.001, *N* = 39 healthy volunteers, Study 1.

Mean ratings for pre-task PA were “dull/tired” to “pleasant/fresh.” Mean ratings for post-task PA were “cheerful/lively” to “delighted/energetic” (Figure 1). This pattern of responses indicates a shift in the sample from a relative absence of motivationally charged positive emotion (i.e., sluggish lethargy) to the activation of incentive motivation, which at its high-end manifests as exuberant energy (see Methods). Task-induced PA was significant [ANOVA task main effect, *F*(1,38) = 55.3, *p* < 0.001] and large in effect size (*d* = 1.7), with a large task-induced rise in PA on both study days [pre- vs. post-task *t*-tests: day 1 (*t*(38) = 6.0, *p* <0.001, *d* = 1.1); on day 2 (*t*(38) = 6.3, *p* < 0.001, *d* = 1.3)]. There was no main effect of day (*F*(1,38) = 3.37, *p* = 0.07) and the task by day interaction for PA was not significant (*F*(1,38) = 0.02, *p* = 0.88), indicating task-induced PA did not differ between day 1 and day 2 (*t*(38) = −0.15, *p* = 0.88). These findings indicate a large, reproducible task effect on PA that did not differ across study days.

Mean ratings for pre-task NA were “calm” to “relaxed.” Mean ratings for post-task NA were “annoyed” to “tense” (Figure 1). This pattern of responses indicates a shift in the sample from relaxation to annoyance, signifying task-induced elicitation of negative agentic emotion (see Smith and Ellsworth, 1985; Shaver et al., 1987; Harmon-Jones et al., 2010). Task-induced NA was significant [ANOVA task main effect, *F*(1,38) = 44.7, *p* < 0.001] and large in size (*d* = 1.53), with large task-induced rise in NA on both study days [pre- vs. post-task *t*-tests on day 1, (*t*(38) = 4.5, *p* < 0.001, *d* = 0.96); on day 2, (*t*(38) = 6.3, *p* < 0.001, *d* = 1.4)]. The task by day interaction for NA was also significant (*F*(1,38) = 4.2, *p* = 0.049), with greater task-induced NA on day 2 than day 1 (*t*(38) = 2.04, *p* = 0.049). These findings indicate a large, reproducible task effect on NA that was greater on day 2 than on day 1 for the sample.

#### Hypothesis 2. Interrelationship of task-induced exuberance and anger

**Figure 2:**
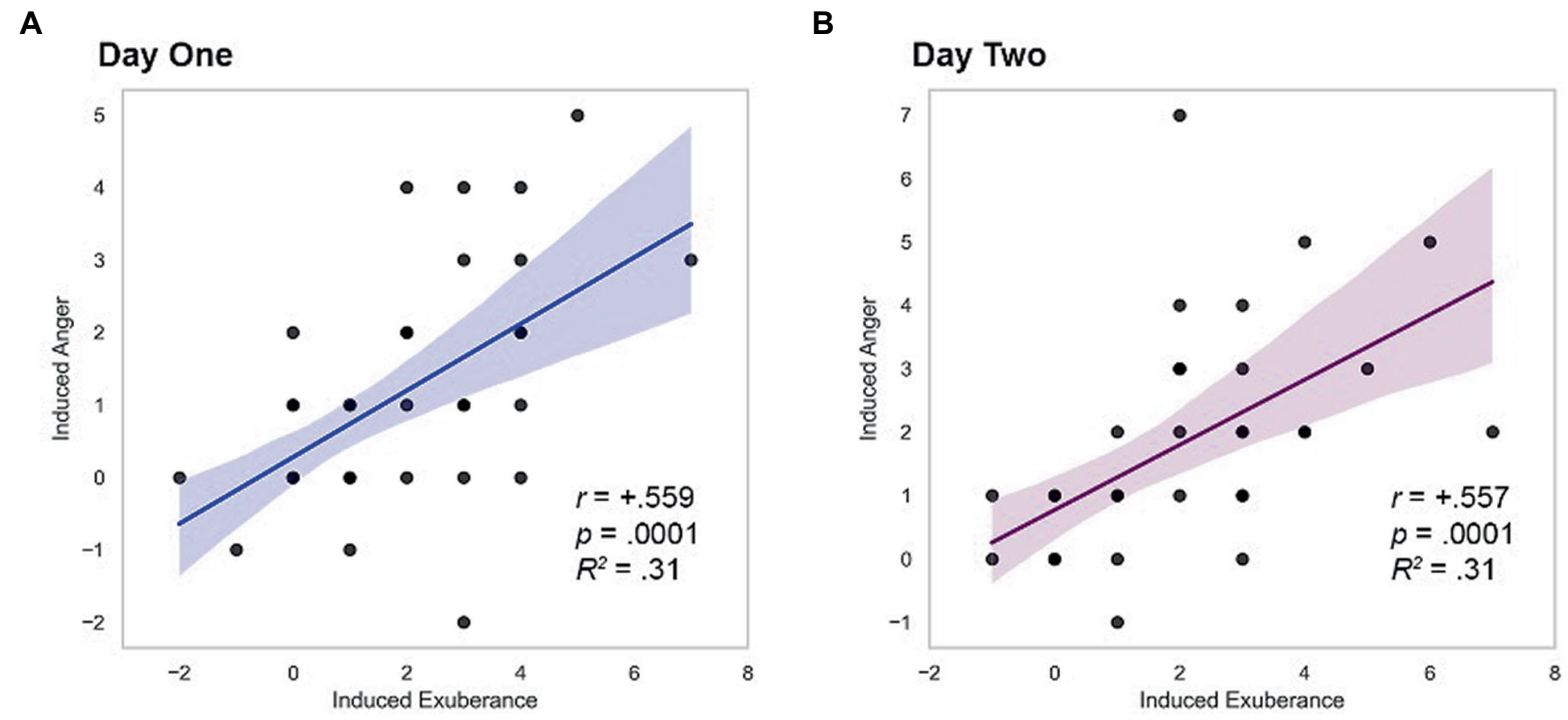
Within-person coherence of induced exuberance and anger. **(A)** Correlation on day one, *r* = +0.559, *p* < 0.001, *R^2^* = 0.31. **(B)** Correlation on day two, *r* = +0.557, *p* < 0.001, *R^2^* = 0.31. Induced exuberance = Post-task PA minus pre-task PA. Induced anger = Post-task NA minus pre-task NA. Task = Incentive Balloon Analogue Risk Task (i-BART). *N* = 39 healthy volunteers, Study 1.

Induced PA and NA correlated positively on both study days (day 1 *r* = + 0.559, *p* < 0.001; day 2 *r* = + 0.557, *p* < 0.001), a large effect (*d’s* = 1.34). These relationships are illustrated in Figure 2.

#### Hypothesis 3. Voluntary approach of risky rewards

Task-induced NA correlated positively with the primary measure of risk-taking on both days (day 1 *r =* 0.27, *p* = 0.047; day 2 *r* = 0.36, *p* = 0.01), and the secondary measure of balloon explosions on both days (day 1 *r* = 0.33, *p* = 0.02; day 2 *r* = 0.37, *p* = 0.01), and the secondary measure of monetary earnings on day 2 when the task was well-learned (day 1 *r* = 0.25, *p* = 0.06, n.s.; day 2 *r* = 0.34, *p* = 0.018). Significant effects were medium in size (*d* = 0.56 to 0.77), and survived Bonferroni correction for multiple comparisons (Table 2). In contrast, findings for induced PA were mixed, with null findings on day 1 and trend-level to positive relationships on day 2 (primary measure: risk behavior: day 1 *r* = *−*0.11, *p* = 0.26; day 2 *r* = *+*0.23, *p* = 0.08; secondary measures: balloon explosions: day 1 *r* = −0.01, *p* = 0.49; day 2 *r* = *+*0.26, *p* = 0.05; monetary earnings: day 1 *r* = −0.03, *p* = 0.43; day 2 *r* = +0.16, *p* = 0.17). The significant finding for PA (induced PA and balloon explosions on day 2) was medium in size (*d* = 0.54, Table 2), and did not survive Bonferroni correction for multiple testing (adjusted alpha = 0.025).

**Table 2:** Induced exuberance, anger and volitional approach of risky reward.

	Induced exuberance		Induced anger	
	Day 1 *r (p)*	Day 2 *r (p)*	Day 1 *r (p)*	Day 2 *r (p)*
**Primary measure**				
Risk behavior	−0.11 (0.26)	0.23 (0.08)^+^	0.27 (0.047)*	0.36 (0.01)**
**Secondary measures**				
Balloon explosions	−0.01 (0.49)	0.26 (0.05)* ^**#**^	0.33 (0.02)*	0.37 (0.01)**
Money earned	−0.03 (0.43)	0.16 (0.17)	0.25 (0.06)^+^	0.34 (0.02)*

Task-induced anger was significantly positively related to risk behavior and balloon explosions on both study days, and monetary earnings on the i-BART task on day two. Significant findings with NA survived Bonferroni correction for multiple comparisons (alpha = 0.05 for primary measures; adjusted alpha = 0.025 for secondary measures, see methods). Task-induced exuberance was positively related to balloon explosions on day two. Relationship with PA did not survive correction for multiple comparisons (adjusted alpha = 0.025). Induced exuberance = post-task PA minus pre-task PA. Induced anger = post-task NA minus pre-task NA. ^+^*p* < 0.10; *p ≤ 0.05; **p = 0.01; ^#^n.s. after Bonferroni correction for multiple comparisons. *N* = 39 healthy volunteers, Study 1.

#### Hypothesis 4. Relationship with trait agency

**Figure 3:**
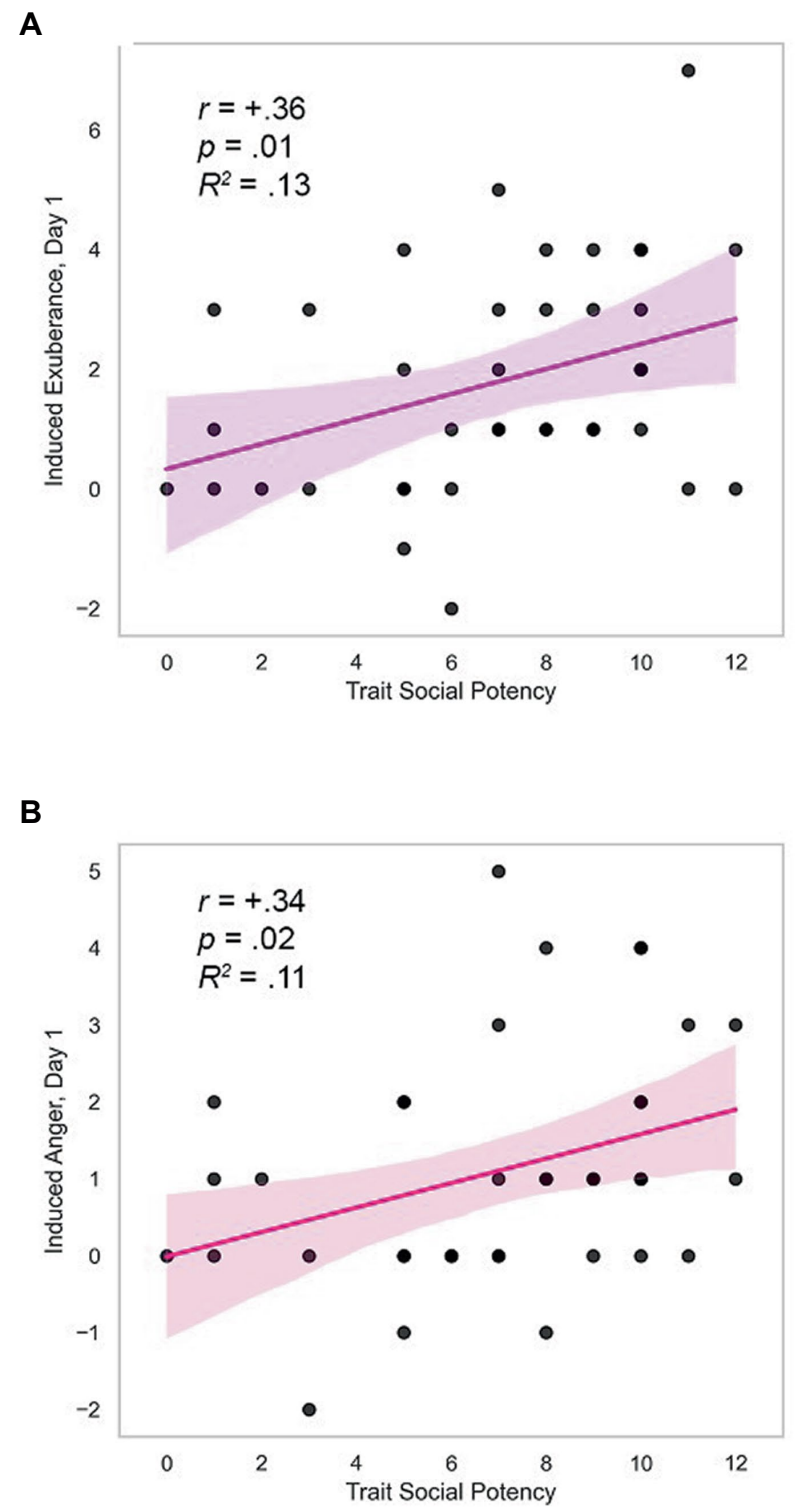
Trait SP predicts induced exuberance and anger. **(A)** Correlation of trait SP with induced exuberance on day one, *r* = +0.36, *p* = 0.011, *R*^2^ = 0.13. **(B)** Correlation of trait SP with induced anger on day one, *r* = +0.34, *p* = 0.018, *R*^2^ = 0.11. Induced exuberance = Posttask PA minus pre-task PA. Induced anger = Post-task NA minus pre-task NA. Task = Incentive Balloon Analogue Risk Task (i-BART). *N* = 39 healthy volunteers, Study 1.

*Day 1.* Trait SP (agency) was positively correlated with task-induced PA and NA on day 1 (induced PA_day1_: *r* = +0.36, *p* = 0.011, *R^2^* = 0.13; induced NA_day1_: *r* = +0.34, *p* = 0.018, *R^2^* = 0.11; Table 3). These effects were medium in size and are illustrated in Figure 3. Induced PA was positively related with trait affiliative extraversion (trait SC: *r* = +0.34, *p* = 0.035), negatively related with trait anxiety (trait SR: *r* = −0.32, *p* = 0.044), and unrelated to other measures (*r* ≤ |0.17|, *p* > 0.30, Table 3). Follow-up analyses indicated that induced PA was positively related to SP after contributions of SC and SR were accounted for (partial *r* = +0.28, *p* = 0.046, *N* = 39). Task-induced NA was not associated with other traits (*r’s* ≤ |0.27|, *p’s*≥0.10, Table 3). These data indicate specificity of induced PA and NA to trait SP. *Day 2.* In contrast, there were no differences in induced PA and NA by personality on day 2 (*r* ≤ |0.20|, *p*≥0.11; details in Supplementary Table 1).

**Table 3:** Agentic extraversion predicts induced exuberance and anger.

Personality traits	Induced exuberance Day 1 *r (p)*	Induced anger Day 1 *r (p)*
**Primary measure (agentic)**		
*Trait agency*		
Social potency	0.36 (0.011)**	0.34 (0.018)*
**Discriminant measures (non-agentic)**		
*Trait anxiety*		
Stress reaction	−0.32 (0.04)*^#^	0.08 (0.62)
*Trait impulsivity*		
Control	−0.11 (0.52)	−0.001 (0.99)
*Trait affiliation*		
Social closeness	0.34 (0.035)*^#^	0.12 (0.46)
*Trait fear/cautious timidity*		
Harm avoidance	−0.02 (0.91)	0.08 (0.61)
*Trait immersive emotion*		
Absorption	−0.16 (0.33)	0.27 (0.10)*^+^*
*Trait interpersonal aggression*		
Aggression	−0.17 (0.32)	0.23 (0.16)

Induced exuberance and anger to the task on day 1 (i.e., initial exposure to the stimulus) was related to participants’ scores on trait social potency (SP), and was unrelated to other, non-agentic traits measured in the same individuals. Relationships with non-agentic traits did not survive correction for multiple comparisons (adjusted alpha = 0.008). Induced exuberance = post-task PA minus pre-task PA, Induced anger = post-task NA minus pre-task NA (details in methods). Task = i-BART. Personality measures on the Multidimensional Personality Questionnaire Brief Form (MPQ-BF). **p ≤ 0.01, *p ≤ 0.05, ^+^*p* ≤ 0.10, ^#^n.s. after Bonferroni correction for multiple comparisons. *N* = 39 healthy volunteers, Study 1.

#### Hypothesis 6. Convergent validity

The task increased participants’ ratings on VAS Elation (*t*(37) = 1.75, *p* = 0.04), a small effect (*d’s* = 0.18). Task-induced VAS elation was significantly positively related with task-induced rise in PA and NA (Induced PA_day1_ *r* = 0.37, *p* = 0.011; Induced PA_day2_ *r* = 0.32, *p* = 0.025; Induced NA_day1_ *r* = 0.35, *p* = 0.015; Induced NA_day2_ *r* = 0.37, *p* = 0.011), all medium effects. These medium effects are illustrated in Supplementary Figure 1 and provide modest evidence of construct and convergent validity.

#### Hypothesis 7. Divergent validity and state specificity

Six non-agentic states were evaluated to provide information on specificity and divergent validity. The task increased participants’ ratings on POMS Arousal (*t*(37) = 2.1, *p* = 0.04), a small effect (*d’s* = 0.18). This effect did not survive Bonferroni correction (adjusted alpha = 0.008). There were trend-level task effects on diastolic and systolic BP (diastolic BP *t*(38) = 2.0, *p* = 0.06; systolic BP *t*(38) = 1.73, *p* = 0.09); these did not survive Bonferroni correction. There were no task effects on HR or anxiety (HR *t*(38) = 1.0, *p* = 0.31; POMS Anxiety *t*(37) = −0.28, *p* = 0.78; VAS Anxiety *t*(37) = 0.45, *p* = 0.65). Follow-up analyses indicated physiological arousal, subjective arousal, and subjective anxiety were not related to task-induced rise in PA or NA (*r’s* ≤ |0.27|, *p’s*≥0.10, details in Supplementary Table 2). These findings provide robust evidence of discriminant validity and specificity of task effects on PA and NA.

#### Hypothesis 8. Divergent validity and trait specificity

Induced exuberance and anger to the task on day 1 (i.e., upon initial task exposure) was largely unrelated to non-agentic traits, such as trait anxiety (measured by MPQ-BF Stress Reaction), trait impulsivity (measured by MPQ-BF Control), trait affiliation (measured by MPQ-BF Social Closeness), trait fear/cautious timidity (measured by MPQ-BF Harm Avoidance), trait immersive emotion (measured by MPQ-BF Absorption), and trait interpersonal aggression (measured by MPQ-BF Aggression). While Stress Reaction and Social Closeness were positively associated with task-induced exuberance on day 1 (Table 3), these relationships did not survive Bonferroni correction for multiple tests (adjusted alpha = 0.008). These data indicate there was no significant relationship between task-induced anger and non-agentic traits (Table 3).

#### Hypothesis 9. Predictive validity

The main effect of the task on PA (above) indicates stability and test–retest validity of induced PA over time and was unqualified by day or interaction effects. In contrast, for NA the main effect of task and the task x day interaction effect (above) were significant, indicating a rise in induced NA over time (above). These findings provide evidence for behavioral sensitization of induced NA over time.

### Study 1 discussion

Findings were largely consistent with the tenets of the agentic model. Given the modest sample size and the number of tests conducted, findings in study 1 are considered preliminary. Our findings were as follows. The incentive task increased both PA and NA in the same individuals (tenet T1, Table 1). Effects were large in size, and task-induced rise in PA correlated with rise in NA, indicating a coordinated rise in both responses (tenet T2). Induced NA had behavioral consequences, relating to the voluntary approach of risky rewards on both study days (tenet T3). Induced PA and NA to the task at first exposure (day 1) was strongly positively related to SP, a measure of trait sensitivity to reward (tenet T4). There was concordance between induced PA, NA and other positively valenced agentic states, such as elation (tenet T6). There was state and trait evidence of discriminant validity. Induced NA was unrelated to non-agentic states, indicating independence from other states such as anxiety (tenet T7). Induced NA was unrelated to non-agentic traits such as trait impulsivity, aggression, anxiety, affiliation, fear, and immersive emotion, indicating specificity to trait SP (tenet T8). Regarding predictive validity (tenet T9), there was consistency in agentic responses over time, with potential sensitization of agentic anger by day 2. The greater task-induced NA on day 2 than day 1 is consistent with behavioral sensitization of other incentive phenomena in healthy individuals (Depue and Collins, 1999; Depue and Fu, 2013). While preliminary, study 1 findings are largely aligned with the tenets of the agentic model (Table 1).

We move now to experiment two, which evaluates model tenet T5 (Table 1), providing preliminary information on potential neural mechanisms.

## Experiment 2

### Rationale and methods

Study 2 evaluated state emotion to the iBART risk task using functional magnetic resonance imaging (fMRI) in in healthy young adult volunteers. The study provides information on neural circuits involved in agentic anger and exuberance responses, relevant to tenet T5 and hypothesis H5 (Table 1). Blood oxygenation level dependent (BOLD) responses to the i-BART task were evaluated in a repeated-measures, within-subjects, placebo-controlled drug challenge design, providing information on VS response to incentive stakes during pharmacological activation of peripheral and central CA. Three levels of stakes (LS, MS, HS) of i-BART were presented in a boxcar design to maximize power to detect effects with fMRI (Friston et al., 1999). Structural and functional MRI were conducted 90 min after administration of placebo (PBO) and *d-*amphetamine (AMP), with a washout period of 48 h between sessions. AMP provides pharmacologic challenge of catecholamine (CA) circuits, providing information on neural reactivity of central and peripheral CA in healthy volunteers (Sulzer et al., 2005; White and Gonsalves, 2020). This AMP input is similar to DA positive prediction error signal, yielding supranormal DA stimulation at postsynaptic receptors in striatum that is not compared with or corrected by reward predictions (Schultz, 2016a). As DA prediction error signal serves “an important function in economic decisions because it helps to update the value signals for the different choice options” (Schultz, 2016a), AMP provides a useful proof-of-concept manipulation to evaluate contributions of CA circuitry in agentic anger responses to risky rewards. Behavioral effects of AMP during the iBART are known and indicate behavioral disinhibition under AMP in males with high trait agency (White et al., 2007). Emotion measures were identical to study 1. VS was evaluated as an area of *a-priori* interest given the role of DA prediction error and risk signal in the region (Schultz, 1998; Fiorillo et al., 2003; Schultz, 2015, 2016b), and our hypothesis that CA processes contribute to agentic anger along a continuum (Table 1).

### Participants

Ten (*N* = 10) healthy, psychostimulant-naïve young adult males were recruited from Brown University and surrounding community. This proof-of-concept pilot was restricted to males, as AMP-facilitated behavioral disinhibition is more readily observed in males than females (White et al., 2007). Inclusion criteria were age 18–35 years, minimum high school education, fluency in English, and being within 20% of ideal body weight. Exclusion criteria for Study 2 were the same as Study 1, with additional exclusions for MRI contraindications (e.g., claustrophobia and bodily ferromagnetic materials). All participants provided written informed consent. The research was approved by the Institutional Review Board at Memorial Hospital of Rhode Island (MHRI) and the Institutional Review Board at Brown University.

### Experimental design

Participants took part in a two-session, within-subjects, double-blinded crossover study of fMRI responses to PBO and a 20 mg dose of oral AMP, a moderately high dose that is well tolerated by healthy volunteers (for discussion, see White et al., 2006b, 2007; Weyandt et al., 2018). Subjects consumed a light meal (bagel, no dairy, no acidic juices) 1 h prior to each session. Subjects received practice on the i-BART and self-report measures prior to MRI imaging. fMRI sessions were conducted during the same time of day between 9 AM and 6 PM to control for circadian effects. Test sessions were 5.5 h apiece, with mood and cardiovascular assessments conducted at half hour intervals outside the MRI scanner to assess and monitor emotional, physiological, and medical status. Participants were confirmed drug-free via urine screening prior to capsule administration each day. Participants were paid $150 for their study involvement, with an additional $20 – $40 earnings possible on the i-BART.

### Drug procedures and dosing

AMP (*d*-amphetamine, Dexedrine^®^, 20 mg oral) tablets were administered in opaque, colored gelatin capsules (size 00) with dextrose filler. PBO was administered in identical gelatin capsules and contained only dextrose. A 20 mg oral dose was selected as it is a general challenge of catecholamine (CA) circuits, providing information on neural responses during activation of central and peripheral CA. At this dosage AMP reliably induces stimulant effects, is well tolerated, and is among the narrow range of doses used with fMRI (Martin et al., 1971; Foltin and Fischman, 1991a,b; Mattay et al., 2000, 2003; Drevets et al., 2001; Uftring et al., 2001; Hariri et al., 2002; White et al., 2006b, 2007, 2018; Kirkpatrick et al., 2013; Langenecker et al., 2020). Pharmacologically, AMP blocks and reverses CA transporters, releasing newly synthesized CA from the cytoplasm of neurons and blocking reuptake of CA from the synaptic cleft (Sulzer et al., 2005; White and Gonsalves, 2020). CA activation is involved in the stress response and experimental administration of CA precursors, such as tyrosine, increase anger states in response to severe psychological stressors (Lieberman et al., 2015). Study 2 provides insight on CA-modulation of VS reactivity during risk approach, relevant to tenet T5 of the agentic model (Table 1). The study thus provides novel information on the intrinsic reactivity of neural substrates supporting agentic anger, incentive reward, and action selection.

### MRI acquisition

Whole brain echoplanar fMRI was conducted in two sessions in each participant using a Siemens 1.5 tesla scanner (TR = 3,860 ms, TE = 40 ms, FOV = 192^2^, Matrix = 64^2^). Sufficient 3 mm thick axial slices were obtained to allow whole-brain coverage. This procedure yielded 68 whole brain volumes per run, for a total of 272 volumes over the four-run acquisition, with a spatial resolution of 3 mm^3^ per voxel. Prior to functional scanning, a whole-brain T1-weighted high-resolution MPRAGE volume was obtained for anatomical reference. MR scanning coincided with the peak period of emotional and physiological response to AMP, 90 min after drug administration (White and Gonsalves, 2020).

### fMRI protocol

**Figure 4:**
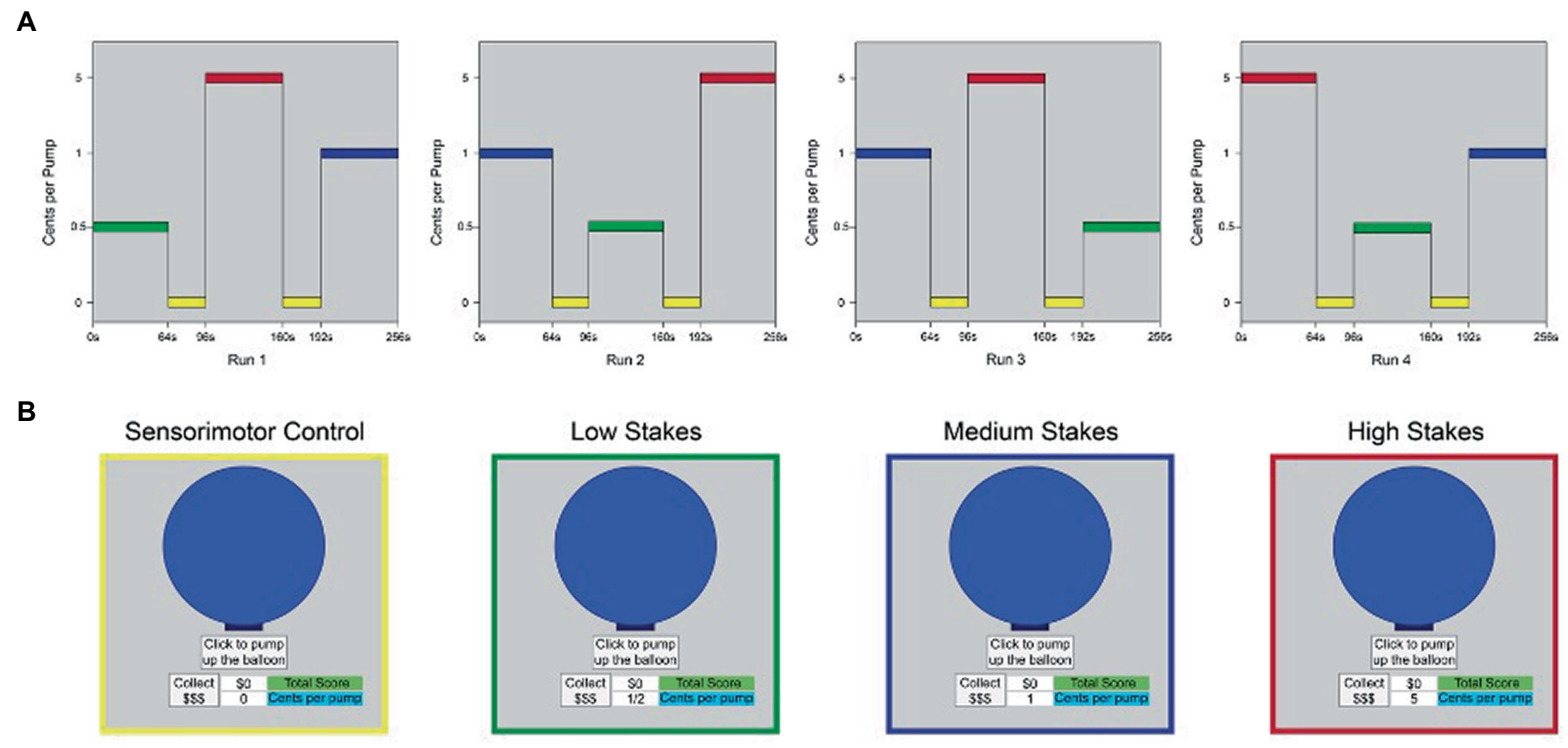
Boxcar design, fMRI iBART task. **(A)** The fMRI i-BART was presented in a boxcar design in four runs. Each run was 262.5 s (4.37 m.) in duration and contained three task blocks of 64 s duration apiece [low stakes (LS), medium stakes (MS), and high stakes (HS)], separated by two sensorimotor control blocks (0 cents/pump) of 32 s duration apiece. Total acquisition time was 17.5 min of functional imaging per session. *Y*-axis indicates incentive value of each block (cents per pump). L = low stakes (0.05 cents/pump), M = medium stakes (1 cent/pump), H = high stakes (5 cents/pump), c = sensorimotor control blocks (0 cents/pump). Values on *y*-axis are not to scale. **(B)** Example of computer screen presented to participants during performance of trials from (left to right): sensorimotor control blocks, low stakes blocks, medium stakes blocks, and high stakes blocks. Images in yellow, green, blue and red outline as in panel **(A)**. Note all trials of the task used a gray-only background; color corresponds to condition in panel **(A)**. *N* = 10, Study 2.

The fMRI i-BART was presented in a boxcar design in four runs, for a total of 17.5 min of functional imaging per session. Each run was 262.5 s (4.37 m) in duration and contained three task blocks of 64 s duration apiece [low stakes (LS), medium stakes (MS), and high stakes (HS)], separated by two sensorimotor control blocks (0 cents/pump) each of 32 s duration. Order of LS, MS and HS incentive blocks were counterbalanced across runs, with individual runs separated by one-minute rest periods (details in Figure 4). Visual stimuli were back projected onto a screen and viewed through a mirror reflection system in the bore of the scanner. Participants heard task explosions through MRI-compatible headphones and pumped up the balloon using an MRI-compatible piano key box with their dominant (right) hand. Within each block, trials were programmed by the first author (TLW) to have a 5-trial running average explosion point of 64 pumps, with a range of explosion points from 1 to 128 pumps, in keeping with laboratory and field versions of the task (Lejuez et al., 2002; White et al., 2007). A fixed order of explosion points was used within each block to reduce variability in presentation. During the sensorimotor control blocks, participants finger-pressed to a $0 condition of the task (0 cents/pump). The control condition provided identical visual and motor stimulation, without an incentive component, providing a conservative control. The study differs from prior studies that focus on fMRI outcomes in clinical patients, other study drugs, and task variants that involve imaginary earnings, single levels of reward, passive behavioral strategies, and assessment outside the scanner (van Eimeren et al., 2009; Claassen et al., 2011).

### GLM and fMRI modeling

Analysis of Functional NeuroImages (AFNI) software (Cox, 1996) was used for fMRI data processing and analyses and SPSS version 25 to evaluate hypothesis 5, relevant to model tenet 5 (Table 1). No participant exhibited excessive motion (≥4 mm) on either fMRI test session (20 sessions, all participants retained). Concatenated 3D + time datasets were temporally smoothed and spatially registered to minimize movement artifact. This procedure yields individual movement correction parameters used as covariates in the general linear model (GLM). To identify the degree to which the incentive blocks elicited responses that differed from the sensorimotor control blocks, first-level analyses of individual brain responses were conducted using GLM with regressors representing the temporal pattern of each condition (LS, MS, HS), including hemodynamic transitions convolved with a gamma function. Modeling using a boxcar design maximizes power to detect effects in fMRI data (Friston et al., 1999). We applied nuisance regressors to account for motion (X, Y, Z, roll, pitch, yaw), with sensorimotor control blocks (0 cents/pump condition) in the baseline, and the GLM incorporated linear and quadratic trends. Following this procedure, functional datasets were co-registered to the high-resolution T1 anatomical dataset, and transformed into standard Talairach stereotaxic space (Talairach and Tournoux, 1988). The data were spatially smoothed using a three-dimensional 6 mm full width at half maximum (FWHM) Gaussian filter, excluding non-brain voxels. Individual t-statistics were generated for each condition per voxel and were used in group-level region of interest (ROI) analyses.

### Study measures and tests of hypotheses

#### State measures of agency

Emotional reactions to the iBART were assessed using participants’ ratings on the 10-point rating scales of PA and NA on the PBO day (Morrone et al., 2000; Weyandt et al., 2018). Participants rated PA and NA scales “right now” 10 min prior to and 10–30 min following MRI imaging. Ratings on the PBO day provide data on global task-induced change in PA and NA, as distinct from specific change in PA to cash-out events and NA to explosion events assessed in study 1.

#### Trait measure of agency

Emotional traits were evaluated using the Multidimensional Personality Questionnaire Brief Form (MPQ-BF) (Patrick et al., 2002a), on a separate in-person screening day prior to MRI. Trait SP provided an index of reward sensitivity (Patrick et al., 2002a), as in study 1. Mean scores on trait SP were 7.5 (s.e. = 0.72) and scores ranged from the 49th – 66th percentile (Patrick et al., 2002b), providing information on the mid-range of scores for the trait.

#### fMRI measure of agentic circuit reactivity

*Rationale*. The nucleus accumbens was selected as an empirical *a priori* region-of-interest (ROI) for evaluation of agentic circuit activity related to anger and exuberance reactions (hypothesis 5, Table 1). The nucleus accumbens is a neural hub that connects motivation and action, and is involved in action selection, behavioral reward, and drug-, cue-, and food-related craving (Depue and Collins, 1999; Fernandez-Espejo, 2000; Floresco, 2015). While value, motivation and salience can be difficult to discriminate because they covary in most situations (Bissonette and Roesch, 2016), reward-related and motor-related activity are processed in human striatum in a ventromedial to dorsolateral (diagonal) gradient (with ventral-medial more reward-related; for discussion see Bissonette and Roesch, 2016). Moreover, as different populations of neurons in the nucleus accumbens encode value and motivation, the region serves as both a ‘limbic-motor interface’ and as a ‘critic’ in ‘actor-critic’ models of reinforcement learning (Bissonette and Roesch, 2016). Given this role we focused our analysis on the nucleus accumbens, providing proof-of-concept information on agentic processing in healthy young adults.

**Figure 5:**
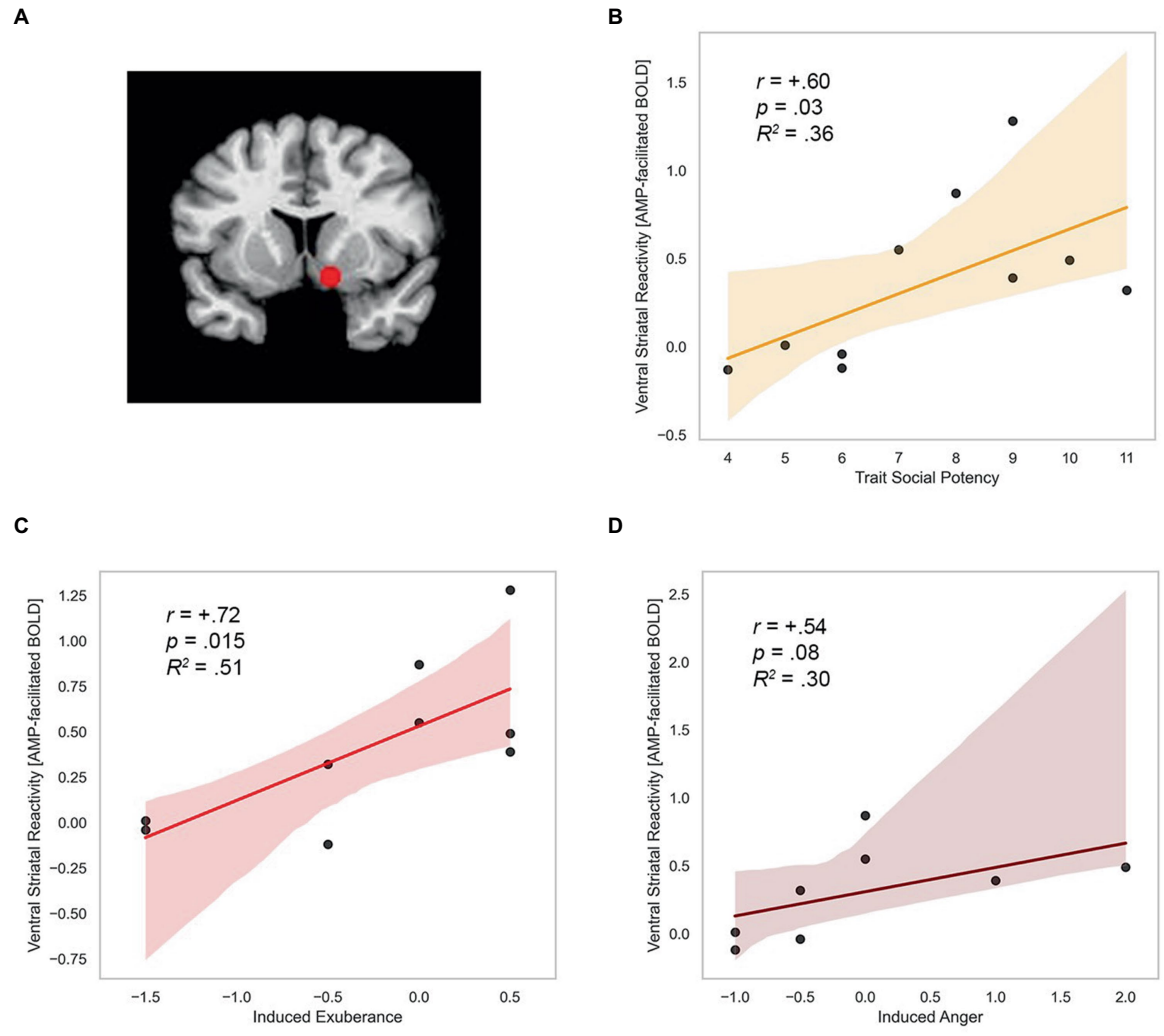
Trait SP, induced exuberance and anger predict ventral striatal reactivity. **(A)** Nucleus accumbens ROI (RAI coordinates +/−12, −8, −8). **(B)** Trait SP predicts CA-modulation of incentives in VS (*r* = +0.60, *p* = 0.03, *R^2^* = 0.36, *N* = 10). **(C)** Induced exuberance and CA-modulation of incentives in VS (*r* = +0.72, *p* = 0.015, *R^2^* = 0.51, *N* = 9). **(D)** Induced anger and CA-modulation of incentives in VS (*r* = +0.54, *p* = 0.08, *R^2^* = 0.30, *N* = 8). SP = Trait Social Potency. Positive Activation = PA. Negative Activation = NA. Induced exuberance = Post-task PA minus pre-task PA on placebo (PBO) session. Induced anger = Post-task NA minus pre-task NA on PBO session. AMP-facilitated BOLD = fMRI response to catecholamine agonist *d*-amphetamine (20 mg; details in methods). *N* = 10 males, Study 2.

*ROI localization*. Mean activation was extracted within two 5 mm radius spheres (515 μL) surrounding the standard bilateral coordinates of the nucleus accumbens in the Talairach Daemon atlas distributed with AFNI (center^2^ of mass RAI coordinates *x* = +/−12, *y* = −8, *z* = −8). Using this approach we extracted mean ROI signal from a standard volume and number of voxels in each participant for each condition of the experiment. This approach provides a consistent brain volume to compare across participants, an effective approach in prior work on drug and reward cues (Xu et al., 2014). The spherical ROI for the left and right nucleus accumbens are overlaid on a standardized template brain in Figure 5A.

*Validity Checks*. Group-level effects were evaluated to verify drug- and stakes-effects in VS, prior to data reduction. Statistical analyses were conducted using mean standardized t-scores per ROI as the dependent measure of brain activity. This approach provided a uniform measure across participants and conditions suitable for parametric analyses. Given prior findings of right-lateralization of approach-related BOLD and left-lateralization of craving-related BOLD (Gordon, 2016), we evaluated right and left nucleus accumbens as separate ROIs to verify drug effects and volitional motor-related activation. Average activity in right and left ROI was extracted in each condition, with drug and stakes effects on BOLD activity verified using a within-subjects, repeated measures drug (2 levels: AMP, PBO) x stakes (3 levels: LS, MS, HS) ANOVA in SPSS (IBM, v25), with Greenhouse–Geisser correction applied for data violating the sphericity assumption. Follow-up post-hoc tests for significant main effects were performed using Tukey’s LSD test, and follow-up paired-samples *t*-tests were conducted for significant drug x stakes interactions. Note an absence of effects in one hemisphere in this analysis does not indicate that magnitude of effects differs across hemispheres.

*Data Reduction: AMP-facilitated BOLD summary score*. Given verification of drug effects and interactions in the ANOVA data validity step, fMRI BOLD data were reduced to a summary score of AMP-facilitated BOLD response to incentive stakes in the right nucleus accumbens in each participant, for use as proof-of-concept data in the evaluation of hypothesis 5, relevant to model tenet 5 (Table 1). fMRI BOLD data in right nucleus accumbens was reduced to a summary score of AMP-facilitated BOLD response to incentive stakes in VS (“AMP-facilitated BOLD”) in each participant. This measure indexes AMP modulation of fMRI BOLD response in VS to stepped incentives, calculated as high [AMP_HS minus PBO_HS] minus low [AMP_LS minus PBO_LS]. This procedure reduced the fMRI data to a single metric in each participant, suitable for analysis with their reports on the state and trait measures of agentic emotion (hypothesis 5, model tenet T5; Table 1).

*Tests for Hypothesis 5*. The summary score of catecholamine circuit reactivity (‘*AMP-facilitated BOLD’,* above) was evaluated with self-reports of state and trait agency using a correlation approach, providing test of hypothesis 5 (relevant to model tenet 5, Table 1). *Independent Measures*. Task-induced PA on PBO, task-induced NA on PBO, and trait SP were entered as separate independent predictors, to test relationships with induced exuberance, induced anger, and trait agency, respectively. Task-induced agentic emotion was operationalized as the difference between pre- and post-task assessments on PBO, calculated as post-task PA minus pre-task PA, and post-task NA minus pre-task NA. These measures provide information on task-induced PA and NA under non-drug (i.e., natural) conditions in each participant. The relationship of participants’ trait SP, task-induced PA on PBO, task-induced NA on PBO, and AMP-facilitated BOLD response was evaluated using a correlation approach. This analysis provides information on the intrinsic reactivity of catecholaminergic agentic circuits to risky reward, and its relationship with natural (non-drug) stimulus-induced agentic anger and exuberance reactions in healthy individuals.

### Data quality and overall analysis

All participants had valid data on the MPQ-BF, providing complete data on SP (*N* = 10). One participant had partial self-report data and was excluded from analyses of PA and NA (*N* = 9). All measures were evaluated for outliers (visually; Cook’s distance D*_i_* scores). In the nucleus accumbens fMRI data, one high influence point with Cook’ distance D*_i_* > 4/*n* was identified and excluded, yielding a final *N* = 8 for fMRI analyses (Bollen and Jackman, 1990). Directional hypotheses were assessed one-tailed and nondirectional hypotheses were assessed two-tailed at an alpha of 0.05 (Table 1). Predictions in hypothesis 5 were independent and were evaluated using single statistical tests of individual summary scores (i.e., positively valenced agentic emotion assessed by induced PA; negatively valenced agentic emotion assessed by induced NA; trait agency assessed by SP; agentic circuit reactivity assessed by AMP-facilitated BOLD). We report trends at an alpha of 0.10 to reduce the impact of Type II error, which has significant adverse effect on scientific progress within and across fields (Amrhein et al., 2019; White et al., 2021). Given the small sample size, findings in study 2 are considered preliminary and provide proof-of-concept data and effect size estimates for future work.

### Results

#### Validity checks

*Nucleus Accumbens Activity. Right ROI*. The drug x stakes interaction effect was significant [*F*(2,18) = 5.14, *p* = 0.017], and there was a significant main effect of drug on BOLD response [*F*(1,9) = 5.93, *p* = 0.038]. The drug by stakes interaction effect is illustrated in Supplementary Figure 2. As seen in Supplementary Figure 2, BOLD response in right nucleus accumbens was lower for HS than MS on the PBO session [*t*(9) = 3.45, *p* = 0.0035] and LS [*t*(9) = 2.77, *p* = 0.011], consistent with risk aversion to rising stakes under PBO per (White et al., 2007). AMP reversed this pattern, increasing BOLD activation overall with greatest activity during HS. AMP thus eliminated the difference in LS, MS, and HS response, with higher BOLD activation to HS under AMP than PBO [*t*(9) = 2.73, *p* = 0.012]. AMP effects on BOLD response were medium to large in effect size and rose as a function of stakes on the task (*d* = 0.51, 0.70, 1.32 for AMP effect on LS, MS, and HS activity, respectively). Main effects of stakes were not significant due to the significant interaction of drug by stakes activity, which qualified the main effect of stakes in the region (see Supplementary Figure 2). *Left ROI*. The main effect of stakes was significant [*F*(2,18) = 4.94, *p* = 0.019]. Rising stakes were associated with a reduction in BOLD activity, with less activation during HS than MS and LS [PBO HS vs. MS: *t*(1,9) = 3.48, *p* = 0.0035; PBO HS vs. LS: *t*(1,9) = 2.04, *p* = 0.036; AMP HS vs. LS: *t*(1,9) = 1.79, *p* = 0.05]. While overall activity was higher under AMP than PBO, with higher BOLD activation to HS under AMP than PBO [*t*(1,9) = 1.87, *p* = 0.047], the main effect of drug [*F*(1,9) = 1.91, *p* = 0.20] and drug x stakes interaction effect [*F*(2,18) = 1.65, *p* = 0.22] were not significant. These data indicate a significant drug by stakes interaction and drug main effect in the right nucleus accumbens, and a significant main effect of stakes in the left nucleus accumbens. Note the absence of a significant drug by stakes interaction in the left ROI does not indicate that the magnitude of this effect differs statistically between the left and right hemispheres. *Data reduction.* Given verification of drug effects and interactions, fMRI BOLD data were reduced to a summary score of AMP-facilitated BOLD response to incentive stakes in the right nucleus accumbens in each participant, for use as proof-of-concept data in evaluation of hypothesis 5 (below).

#### Hypothesis 5. Agentic circuit reactivity: AMP-facilitated BOLD

Trait and state measures of agentic emotion were positively correlated with agentic circuit reactivity, assessed by AMP-facilitated BOLD summary score in each participant. Trait SP related positively to AMP-facilitated BOLD (*r* = +0.60, *p* = 0.03). This effect was large in size (*d* = 1.5) and explained 36% of the variance in BOLD response in the region. This finding is illustrated in Figure 5B. Task-induced PA on PBO was positively related to AMP-facilitated BOLD (*r* = +0.72, *p* = 0.02). This effect was large in size (*d* = 2.08) and explained 52% of the variance in BOLD response. This finding is illustrated in Figure 5C.

Task-induced NA on PBO was marginally positively related to AMP-facilitated BOLD (*r* = +0.54, *p* = 0.08). This association was large in size (*d* = 1.28) and explained 29% of the variance in BOLD response. These data are illustrated in Figure 5D. Formal test of relationships (Fisher r to z transformation) indicates relationships (BOLD response with SP, PA, and NA) did not differ in magnitude or direction (*z*-scores<0.51, *p*’s > 0.6).

**Figure 6:**
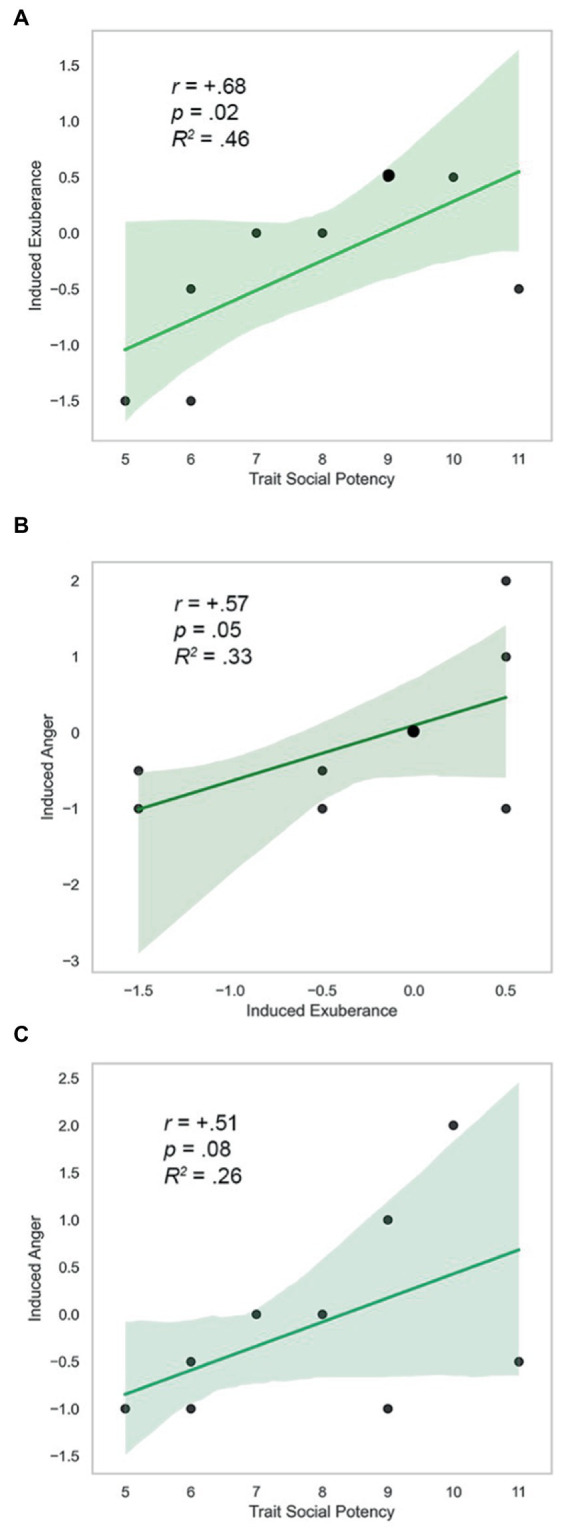
Trait SP, induced exuberance and anger. **(A)** Trait SP and induced exuberance were positively correlated (*r* = +0.68, *p* = 0.03, *R*^2^ = 0.46, *N* = 9). **(B)** Induced exuberance and anger were positively correlated (*r* = +0.57, *p* = 0.05, *R*^2^ = 0.33, *N* = 9). **(C)** Trait SP and induced anger were positively associated (*r* = +0.51, *p* = 0.08, *R*^2^ = 0.26, *N* = 9). SP = Trait Social Potency. Positive Activation = PA. Negative Activation = NA. Induced exuberance = Post-task PA minus pre-task PA on placebo (PBO) session. Induced anger = Post-task NA minus pre-task NA on PBO session. Enlarged datapoints indicate overlapping data from two participants. *N* = 9, Study 2.

Trait SP, task-induced PA on PBO, and task-induced NA on PBO were positively correlated (SP and Induced PA: *r* = +0.68, *p* = 0.02; Induced PA and NA: *r* = +0.57, *p* = 0.05; SP and Induced NA: *r* = +0.51, *p* = 0.08). These findings were uniformly large in size (*d* = 1.9, 1.4, 1.2), and are illustrated in Figure 6. AMP-facilitated BOLD, trait SP, task-induced PA on PBO, and task-induced NA on PBO did not differ in direction or magnitude of relationships (*p’s* > 0.6, n.s.), indicating similar direction and extent of CA-related VS response with trait and state agentic emotion, providing evidence of the coherence of agentic phenomena (model tenet 5) and their predictive validity over time (model tenet 9).

### Study 2 discussion

Study 2 provides novel preliminary data on brain correlates of anger and other valenced agentic emotion. AMP, a general challenge of catecholamine (CA) circuits, provides information on ventral striatal reactivity to stakes during the activation of central and peripheral catecholamine circuits. Trait SP and exuberance related to the direction and magnitude of BOLD response to incentive stakes in right nucleus accumbens, with a similar trend-level effects for anger responses. Task-induced exuberance and anger rose as a function of SP, replicating the results of study 1. Study 2 thus provides preliminary data that largely aligns with expectations of the agentic model of anger and other valenced states (Table 1) and demonstrate VS involvement in agentic phenomena over time (tenets 5 and 9).

## General discussion

**Figure 7:**
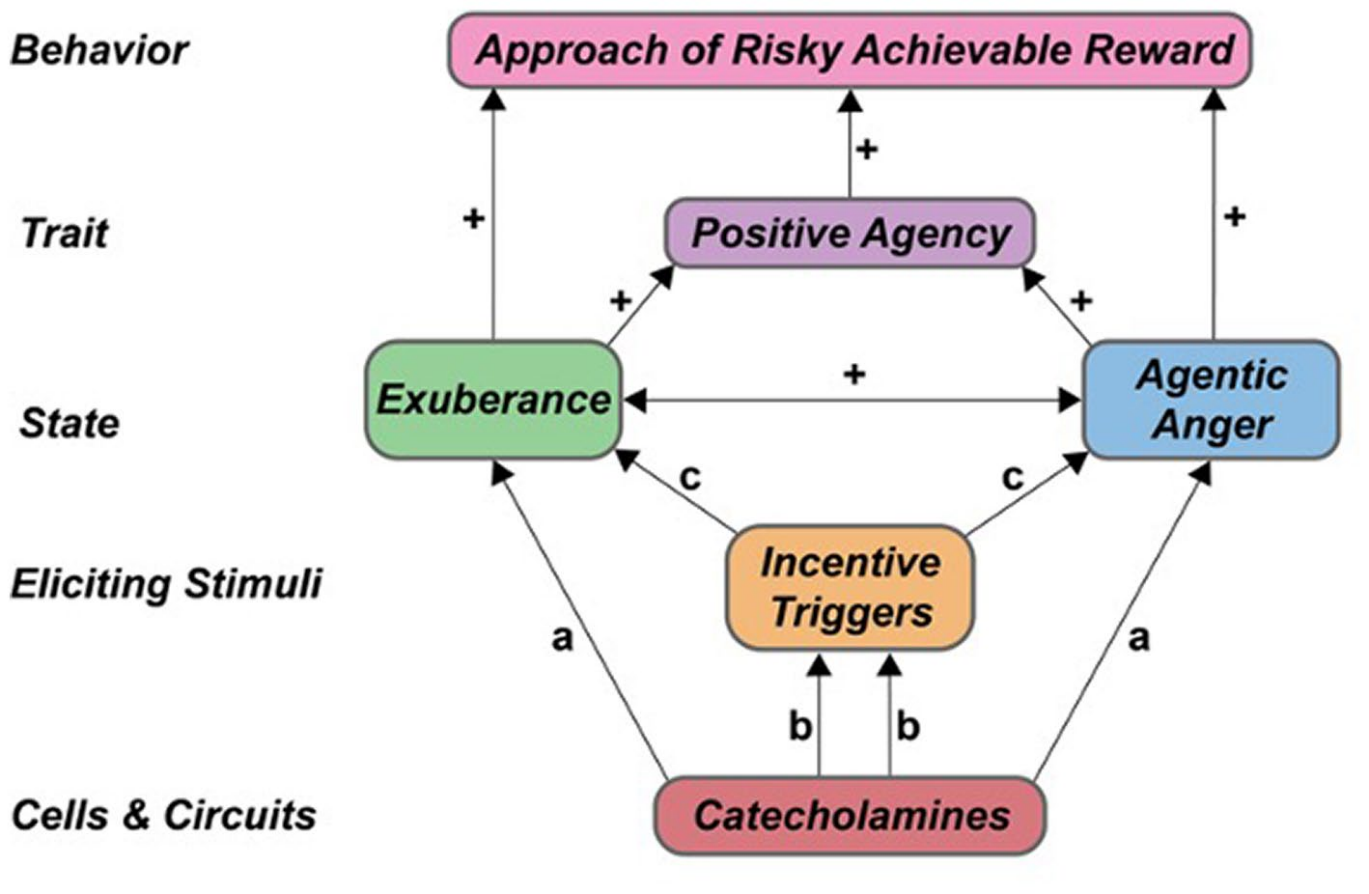
Catecholamine modulation of agentic states, traits and behavior. Proposed contributions of catecholamines to agentic states. ^a^ Denotes direct effects of CA on agentic emotion. ^b^ Denotes CA modulation of perception, processing and reactivity to incentive cues. ^c^ Denotes subsequent effects on agentic states. ^+^ Denotes positive association within-persons. CA, catecholamine.

The above studies provide novel, preliminary proof-of-concept data that anger involves an agentic component. Our findings are consistent with many previous studies that indicate anger differs from other negative emotions, particularly in its underlying motivation. Major tenets of the agentic model of anger (Table 1) were largely supported. Specifically, our incentive challenge task (i-BART) increased exuberance (PA) and agentic anger responses (NA) in both experiments, indicating overlap in the eliciting triggers of valenced agentic states in healthy individuals (tenet T1, Table 1). Agentic anger and exuberance responses during active risk-taking were positively related (tenet T2, Table 1). Participants’ agentic anger and exuberance responses rose as a function of scores on trait SP, indicating contribution of trait reward sensitivity to both states (tenet T3, Table 1). Critically, exuberance and agentic anger responses were also largely unrelated to other traits assessed in the study participants, evidence of discriminant validity. Behaviorally, agentic anger and exuberance each facilitated voluntary approach of risky rewards in the iBART task. These findings are consistent with model tenets T4 and T6, which predict that agentic states – irrespective of valence – should motivate action and voluntary approach of risky rewards (Table 1). Neurobiologically, both trait SP and induced PA related positively to the extent, magnitude and direction of CA-facilitated BOLD response to stakes in the right nucleus accumbens, a region involved in DA-modulation of action selection (Floresco, 2015). These findings provide preliminary evidence of a functional relationship of CA reactivity, right nucleus accumbens activation, induced exuberance, and trait SP (model tenet T5, Table 1). Relationship of CA-facilitated BOLD response and agentic anger induction was similar in direction and smaller in size, indicating greater heterogeneity in the neural correlates of agentic anger, consistent with the complex phenotyping and conceptualization of anger in the larger literature. A schematic providing a visual summary of the agentic model and findings is in Figure 7. Implications for agentic anger, exuberance, risk-taking, persistence and action in real-world contexts are below.

### Agentic stimuli and states

Our incentive task (i-BART) increased agentic states of positive and negative valence (exuberance and agentic anger responses, respectively). Findings were significant and large in effect size in a moderately sized laboratory sample (study 1) and a smaller imaging sample (study 2). Exuberance and anger responses were positively related within and across individuals. Individuals with stronger exuberance responses experienced stronger anger responses, whereas those with less exuberance responses experienced weaker anger responses. This pattern, summarized in Figure 7, indicates task-induced agentic emotion of positive and negative valence are not mutually exclusive and can co-occur in healthy individuals (Beebe-Center, 1965).

### Agentic traits

Exuberance and agentic anger responses were specifically predicted by participants’ trait SP, a measure of trait agency and reward sensitivity, and were – in contrast – wholly unrelated to multiple other traits present in the study participants, including traits of impulsivity, aggression, anxiety, affiliation, fear, and immersive emotion (Table 3 and Figure 7). As trait aggression reflects a predisposition toward hostility and interpersonal conflict with intent to harm, these findings indicate agentic anger can be dissociated from aggression, an important psychometric distinction (White et al., 2021; White and Gonsalves, 2021). Of note, trait SP in healthy young adults has elsewhere been found to relate to gray matter volume in the cingulate gyrus, precentral gyrus, caudate, parahippocampal gyrus, medial orbital frontal cortex and nucleus accumbens (Grodin and White, 2015), and glutamatergic and *N*-acetylated compounds in dACC (White et al., 2021), indicating potential additional targets for future work.

### Agentic circuit reactivity

Study 2 identified the positive relationship of trait SP, exuberance, agentic anger responses, and right nucleus accumbens response to risky decision-making during activation of CA. These findings are consistent with experimental and pharmacologic manipulations that shape negatively valenced agentic responses and risky goal approach through CA signaling. For instance, quinpirole hydrochloride, a D2 receptor agonist, has been shown to facilitate rage and attack in cats (Sweidan et al., 1990; Gregg and Siegel, 2001; Van den Eynde et al., 2008). AMP increases the level of dopamine in the ventral striatum and increases fMRI BOLD response to angry faces in the amygdala in healthy volunteers (Hariri et al., 2002). Sulpiride, a D2 antagonist, has opposing effects and selectively disrupts the ability of healthy men to identify angry faces (Lawrence et al., 2002). Quetiapine, a D2 antagonist, reduces self-reported subjective anger in clinical samples (Gregg and Siegel, 2001; Van den Eynde et al., 2008). Experimental administration of CA precursors, such as tyrosine, increase subjective anger in healthy young adults in response to severe psychological stressors (Lieberman et al., 2015). Anger is also reported elevated in clinical syndromes marked by tonic and phasic alterations in dopamine, such as acute AMP psychosis, drug dependence, bipolar mania, and attention-deficit hyperactivity disorder (Renshaw, 2002; Lara and Akiskal, 2006). This literature suggests specific dopaminergic contributions to agentic anger responses, a possibility that will require assessment using DA-specific manipulations in future work.

Trait SP and state exuberance related positively to ventral striatal reactivity during CA activation, indicating a relationship with action selection (Floresco, 2015). These preliminary data are also consistent with prior work indicating CA facilitation of subjective states of anger during extreme stress in military recruits (Lieberman et al., 2015), and trait SP prediction of reward sensitivity, dopaminergic reactivity, and emotional, behavioral, and neurometabolic responses to psychostimulants in healthy persons (Depue et al., 1994; Depue and Collins, 1999; Morrone-Strupinsky, 2002; White et al., 2006b, 2007, 2018; Depue and Fu, 2013; Jupp and Dalley, 2013; Kirkpatrick et al., 2013). Our findings further suggest trait SP and inducibility of states of exuberance may provide novel markers for the capacity for agentic anger in young adults (Figure 7). These data indicate a role of social leadership in agentic anger experience, and its psychometric independence from aggression, hostility, and intent to harm (Table 3 and Figure 7).

### Agentic behavior

Our preliminary findings indicate agentic anger and exuberance responses predicted the voluntary approach of risky rewards (‘active risk-taking’). It is likely that anger and exuberance engaged distinct proximal and distal processes to facilitate this outcome. Active risk-taking involves four steps – perception of potential reward, risk-taking, and the natural consequences of risk taking, which include the achievement of desired outcomes and/or failure to achieve these outcomes (‘wins’ and ‘blockades’, respectively). Exuberance and agentic anger responses may thus facilitate risk-taking through distinct psychological, emotional, and neural pathways. For instance, exuberance (PA) may foster eager anticipation of potential reward, voluntary approach of reward, and positive reinforcement to wins, creating a positive feedback loop that encourages subsequent risk-taking. In contrast agentic anger responses (NA) may augment the restive anticipation of risky rewards, voluntary approach of risky rewards, positive reinforcement of wins against the odds, and cognitive reframing of adverse consequences’ potential novelty, importance, and salience. Agentic anger and exuberance may thus facilitate voluntary approach (and engagement with) risky rewards (Figure 7) through different antecedent processes, with potential for differential follow-on effects.

Our preliminary data further suggest agentic anger responses relate to DA prediction error signal – the discrepancy between predicted and actual reward – within the VS in healthy individuals (Krigolson et al., 2014). Specifically, agentic anger may relate to DA prediction error signal because anger and other aversive incentive states, in facilitating approach of risky rewards, allows for the emergence of ‘wins’ against the odds. Such wins are (by definition) unexpected, providing a strong positive error signal given the substantive positive difference between received and predicted reward, yielding facilitation of DA-related processing in VS (Stauffer et al., 2016; Schultz, 2016a). Separately, agentic anger may also reduce ‘DA-related negative utility prediction error,’ which reduces synaptic transmission in response to unexpected, non-reward events, i.e., “worse rewards” (Stauffer et al., 2016; Schultz, 2016a). In this way agentic anger may specifically reduce inhibition within VS, increasing volitional action toward reward-related targets. Last, anger-facilitated cognitive reframing of future negative outcomes (i.e., failure or adverse consequences) as less intense, less important, and less novel would reduce the salience of these events, thereby reducing both the likelihood and the magnitude of subsequent DA negative prediction error signals in VS (Gentry et al., 2019). Both processes –anger-related increase in DA positive prediction error, and anger-related reduction in DA negative prediction error– serve to functionally increase VS reactivity to risky reward stimuli, particularly when central and peripheral CA circuits are activated (e.g., during stress, arousal, or conflict; and in response to natural or experimental exposure to CA-modulating drugs such as study 2). These mechanisms explain and are consistent with relationships observed here with trait SP, exuberance, agentic anger, and CA-induced VS responses during risky decision-making (Figures 5–7). Activation of agentic anger may thus engender forward, risky approach that is persistent to adverse consequences via multiple effects on DA prediction error processing in VS. There are real-world implications. For instance, amongst veterans, subjective anger post-deployment predicts risk-taking behavior in the following 4 months (Adler et al., 2011), illustrating a potentially longstanding relationship of agentic anger and risky approach in real-world contexts.

### Societal and clinical implications

Our preliminary data indicate agentic anger responses motivate personal action toward goals that entail risk, consistent with prior concepts of ‘moral anger.’ Informed by traditions of moral psychology, ‘moral anger’ represents a response to perceived harm of another, injustice, unfairness, vulnerability, and norm violations (Gutierrez and Giner-Sorolla, 2007; Dijker, 2010; Russell and Giner-Sorolla, 2011; Brosnan and de Waal, 2014; Lindebaum and Geddes, 2016; O'Reilly et al., 2016). The subjective experience of moral anger is consistently implicated in motivating potentially risky personal actions that would not otherwise be undertaken by an individual (Gutierrez and Giner-Sorolla, 2007; Dijker, 2010; Russell and Giner-Sorolla, 2011; Brosnan and de Waal, 2014; Lindebaum and Geddes, 2016; O'Reilly et al., 2016). In events requiring courage to act on behalf of witnessed injustice, anger consistently predicts intervention on behalf of other people, providing personal “motivational fuel to act” (Sasse et al., 2020). Moral and agentic anger thus have significant overlap, both conceptually and phenomenologically. Current exemplars of moral anger include efforts to curb global warming; Black Lives Matter activism in the U.S. and internationally; efforts to make vaccines equitably available for COVID-19 and other infectious diseases across the globe; and the efficacy of internal states of subjective anger in motivating women to leave domestic abusers (Choi et al., 2009; Maneta et al., 2012). In each case, active search for safety, resolution or remediation can trigger an increase in the risk of personal harm (social, economic, emotional, financial, physical), thereby increasing external barriers to action at the very time such action is required. As noted by Martin Luther King: “We are confronted with the fierce urgency of now…. We must move past indecision to action” (King, 1967; see also King, 1963; Walters, 2021). In two studies of psychologically and physiologically healthy volunteers, we find that states and traits of agentic anger motivate voluntary action toward goals that entail risk. Such processes are relevant to individual and collective action for social justice, equity and human rights (White and Gonsalves, 2021), and complement a large body of work in social and political science indicating “anger is an emotion that mobilizes people to act” (Schaffner, 2022).

Our further finding that agentic anger responses may sensitize over time (task by day interaction, *p* < 0.05) has implications for post-deployment veterans and individuals living with post-traumatic stress disorder (PTSD). In veterans, anger is a response to repeat exposure to trauma (Adler et al., 2011), and is higher in those diagnosed with PTSD than other disorders (Munley et al., 1995). Risky rewards in military contexts are many and include the need to achieve safety, avoid physical harm and potential loss of friends, mentors, and family. Repeat exposure to trauma in military contexts (Adler et al., 2011) requires repeated, risky approach of safety (itself a risky reward). Behavioral sensitization of agentic anger responses may thus provide a novel target for clinical interventions in PTSD, particularly when PTSD is characterized by a preponderance of anger-related phenomenology as opposed to emotions of fear, helplessness, depression or anxiety (Adler et al., 2008).

### Strengths and limitations

The present studies have specific strengths and limitations. The studies provide initial, proof-of-concept data on a novel theoretical model using two carefully conducted study designs, which combine a behavioral economic task with neuroimaging and pharmacology in healthy young adult volunteers. The research strategy is ambitious and includes multiple experiments with multiple visits to the lab, fMRI scanning, and drug administration via a double-blinded, placebo-controlled crossover design. Limitations of both studies include their modest sample size, providing initial, preliminary proof-of-concept data and effect size estimates. Strengths of study 1 include use of a computerized challenge task presenting opportunities to approach achievable, risky rewards (i-BART), providing objective challenge of risky approach behavior and agentic emotion. Study 1 evaluated positively and negatively valenced agentic states at two time points (pre- and post-task) on two sessions in each participant using a within-subjects repeated measures design. This study design provides good power to detect large effects and novel within-subject data on reproducibility, habituation, and sensitization of valenced agentic states in healthy individuals. Multiple measures were included to provide information on construct validity, convergent validity, discriminant validity and specificity. Study hypotheses were directional and independent, with the majority evaluated via single tests (hypotheses 1, 2, 4, 5, 6, 9), an approach that minimizes Type I error. Hypotheses involving multiple tests (hypothesis 3, 7, 8) were corrected for multiple comparisons using the Bonferroni method, a highly conservative correction for Type I error. Strengths of study 2 included evaluation of fMRI BOLD reactivity to stakes for risky decision-making during active, placebo-controlled experimental manipulation of CA in healthy participants. This was accomplished using a within-subjects AMP drug administration procedure in a double blinded, time-locked, PBO-controlled cross-over study design. The use of the boxcar iBART task design maximized statistical and experimental power to detect fMRI effects (Howard et al., 1996; Friston et al., 1999; Mattay et al., 2000; Uftring et al., 2001; Hariri et al., 2002; Knutson et al., 2004; Bell et al., 2005; Sommer et al., 2006; Cowan et al., 2008; van Eimeren et al., 2009; Schouw et al., 2013). Limitations of study 2 were several and included the small sample size, use of a male-only sample, the lower resolution (1.5 T) of acquisition, and evaluation of effects in the nucleus accumbens, an anatomical brain region in the VS with proximity to ventricles and sinuses and potential susceptibility artifact. While the sample was relatively small, the data are unique, extend and replicate the results of study 1, and are complementary to the larger literature on emotion, risk-taking, action selection, DA prediction error, individual differences, and incentive motivation in healthy volunteers. Identified findings in both studies were medium to large in effect size, with a number of significant *p*-values hovering around 0.05. This is to be expected as effect sizes of significant findings scale inversely with sample size and statistical power (Serdar et al., 2021). The present results should be considered preliminary, and require replication in larger, more well-powered follow-up studies. Relationships identified here are also likely to reduce in magnitude as larger samples with greater heterogeneity are evaluated in future work. In addition, while agentic anger may motivate actions, the selection of actions – and their associated consequences – may in turn shape the experience of anger in real-world settings. Future work should thus compare the relative weight and direction of the relationship of anger, selection, action, and consequences. Moreover the context in which agentic anger occurs is likely to affect the timing and extent of risky decision-making. Last, it is likely that there are multiple distinct forms of anger, of which agentic anger is only a subset. Rigorous exploration of the diversity of anger states is thus important in future work to validate, replicate and extend the findings. The present effort provides a forward step in understanding and documenting this diversity.

The present studies, with their given strengths and limitations, provide unique preliminary proof-of-concept data on the agentic properties of anger and its relationship to action in psychologically and medically healthy young people. Agency is a fundamental aspect of human rights and dignity (White and Gonsalves, 2021). These preliminary findings have relevance for motivation of personal and group action in pursuit of social justice: a timeless, difficult and uncertain goal that entails significant risk of harm for activists in a wide range of contexts (Friston, 2012; Bacchetti, 2013; Button et al., 2013; Quinlan, 2013).

### Future directions

Future work on agentic anger and other negatively valenced agentic states (Figure 7) will benefit from inclusion of additional measures such as courage, indignation, righteousness, rage, and moral injury, which were not evaluated in the present studies. Inclusion of such measures will provide important information on the subjective correlates of agentic anger and other negatively valenced states. Contextual factors such as ambient noise, physical discomfort, misinformation, zero-sum thinking, scarcity, threat, exclusion, attributions of causality, unfairness and disrespect may also shape agentic anger responses in real-world settings, and deserve evaluation in future work (Berkowitz and Harmon-Jones, 2004; Clore and Centerbar, 2004). Agentic anger may also affect behavior in a variety of contexts, including financial, social, and physical risk-taking. Evaluation in such settings is thus recommended. The present studies assessed agentic anger along a dimension of intensity using a general measure of negatively valenced activated (agentic) emotion to reduce social desirability bias in participants’ NA responses. Future work can include measures such as the State and Trait Anger Expression Inventory (STAXI-2), which provide broad assessment of subjective anger experience (Spielberger et al., 1999). Evaluation of specific dopaminergic, noradrenergic and glutamatergic contributions to agentic anger and other negatively valenced agentic states are warranted, as AMP provides a nonspecific, general challenge of catecholamine (CA) circuitry in healthy individuals (White et al., 2018; White and Gonsalves, 2020). Additional neuroimaging techniques, such as functional magnetic resonance spectroscopy, can be used to provide relevant information on glutamatergic compounds *in vivo* (White et al., 2018, 2021). Future samples should be powered to detect small to medium effects (see Pernet et al., 2012) to minimize Type I and Type II error, the latter of which has significant adverse impact on scientific progress within and across fields (Amrhein et al., 2019; White et al., 2021). Additional brain regions implicated in positive utility DA prediction error signal should also be evaluated, such as the globus pallidus, subthalamic nucleus, pars reticulata of the substantia nigra, pedunculopontine nucleus and ventral tegmental area (Depue and Collins, 1999; Schultz, 2016b). Last, we would encourage exploration of the ways in which these findings may have relevance to other aversive agentic states – such as craving, boredom, irritation, and frustration – relevant to behavioral dysregulation triggered by (and related to) supranormal, desired rewards, such as drug-, food-, and gambling- related stimuli. The findings may also have relevance to a fuller understanding of agency and anger in real-world contexts, such as Title VII employment discrimination and the legal system (see Carle, 2005, 2016).

### Summary and conclusions

In sum, the present studies provide two lines of preliminary proof-of-concept evidence for the agentic properties of anger and other negatively valenced states in healthy humans (Table 1 and Figure 7). Our dimensional measure of agentic anger (NA) related positively to states of exuberance (PA), trait reward sensitivity (trait SP), and participants’ voluntary approach of risky rewards, consistent with the agentic model. Exuberance and trait SP were positively related to AMP-facilitated rise in ventral striatal BOLD activity to incentive stakes, consistent with known CA contribution to euphoria and anger (Drevets et al., 2001; Lieberman et al., 2015). Our results indicate that the voluntary approach of risky goals can be accomplished through negative as well as positive agentic emotional states in healthy people (agentic anger and exuberance). This pattern of findings suggests an elegant, evolved neurobehavioral solution to the difficult problem of how neurobiological systems can support the ongoing, resilient approach of elusive reward targets in the face of known (or potential) risk, uncertainty, obstacles, harm, or loss. Agentic anger is thus likely to be highly relevant to a wide variety of deeply important human processes, including but not limited to exuberance, joy, persistence, risk-taking, and motivation of decisive personal action and engagement in response to social injustice, inequity, and violations to agency and intrinsic human dignity (White and Gonsalves, 2021). We look forward to the next steps in better understanding this powerful and important emotion.

## Data availability statement

The raw data supporting the conclusions of this article will be made available by the authors, without undue reservation.

## Ethics statement

The studies involving human participants were reviewed and approved by the Institutional Review Board at The University of Chicago (White et al., 2007, 2008) and the Institutional Review Board at Memorial Hospital of Rhode Island (MHRI) and the Institutional Review Board at Brown University. The patients/participants provided their written informed consent to participate in this study.

## Author contributions

TW: conceptualization, data curation, formal analysis, funding acquisition, investigation, methodology, project administration, resources, software, supervision, validation, visualization, writing—original draft, writing—revision, review, and editing. MG: conceptualization, software, visualization, and writing—review and editing. CZ, RC, and AN: conceptualization and writing—review and editing. HJ: visualization and writing—review and editing. UC: data curation, formal analysis, software, and writing—review and editing. LS: data curation, formal analysis, resources, software, and writing—review and editing. CL: conceptualization, methodology, software, and writing—review and editing. All authors contributed to the article and approved the submitted version.

## Funding

This research was supported by grants from the National Institute on Drug Abuse (R03-DA017178 and R21-DA029189 to TW), the Ittleson Foundation for Brain Research (TW), Brown University Center for Alcohol and Addiction Studies Research Excellence Award (TW); National Science Foundation Graduate Research Fellowship (DGE1058262 to AN); predoctoral training grant from the National Institute for Mental Health (T32MH020068 for AN); National Institute on Alcohol Abuse and Alcoholism (T32AA007459 for USC); Carney Institute Graduate Award in Brain Science (MG); and the Zimmerman Fund for Scientific Innovation Awards in Brain Science, Robert J. and Nancy D. Carney Institute for Brain Science (TW). TW has served as scientific advisor and consultant to Strategic Aid Partners, a 501c3 organization (San Francisco, CA).

## Conflict of interest

The authors declare that the research was conducted in the absence of any commercial or financial relationships that could be construed as a potential conflict of interest.

## Publisher’s note

All claims expressed in this article are solely those of the authors and do not necessarily represent those of their affiliated organizations, or those of the publisher, the editors and the reviewers. Any product that may be evaluated in this article, or claim that may be made by its manufacturer, is not guaranteed or endorsed by the publisher.

## Supplementary material

The Supplementary material for this article can be found online at: https://www.frontiersin.org/articles/10.3389/fpsyg.2023.1060877/full#supplementary-material

## Footnotes

^1^Note, hypothesis 5 (model tenet T5) is evaluated in study 2.

^2^ http://www.talairach.org/daemon.html
