# Supplementary material for: Anger, agency, risk and action: a neurobehavioral model with proof-of-concept in healthy young adults
Source: Front Psychol. 2023 May 30;14:1060877. doi: 10.3389/fpsyg.2023.1060877 (PMC10261990; doi:10.3389/fpsyg.2023.1060877)
Supplement: Supplementary file 1 [file Data_Sheet_1.pdf]

Supplementary Material

SI Methods, Study 1

Incentive Balloon Analogue Risk Task (iBART)

The BART is a computerized task on which participants have the opportunity to win or lose potential earnings, where persistent responding increases gains but also increases the risk of loss on each trial. The BART has high test–retest stability (Lejuez et al., 2003; White et al., 2008), making it appropriate for use in repeated measures designs. In this task, participants are presented with “balloons” on a screen, and participants are given the opportunity to “pump” the balloon to earn monetary rewards. On each trial the computer screen displays a small balloon, a balloon pump, a reset button labeled “Collect $$$,” a box displaying the amount of money earned on the previous trial, a box displaying the total amount of money earned, and a box showing how much the balloon currently displayed would pay off (Lejuez et al., 2002). The number of points that can be earned per pump varies across trials, with 20 balloons having low value (0.5 cents per pump), 20 balloons having medium value (1.0 cents per pump) and 20 balloons having high value (5.0 cents per pump). On each trial, the number of cents earned increases with each pump until either (a) the balloon “pops” and participants lose their earnings for that trial, or (b) the participant collects the accumulated earnings for that trial. On each trial, each individual click on the pump inflates the balloon one degree (about .125 inches in all directions), and each balloon is programmed to pop between 1 and 128 pumps, with an average breakpoint of 64 pumps. Specific information regarding the balloon breakpoint determination is not provided to the participants, who are simply informed that the balloon can break anywhere from the first pump all the way through enough pumps to make the balloon fill the screen. At any point during each trial, the participants can stop pumping the balloon and click the “Collect $$$” button, which transfers money accumulated from that balloon to the permanent bank, updates the permanent bank amount on the display, and produces a slot machine payoff sound. In contrast, when a balloon explodes, a “pop” sound is heard, the balloon disappears, the money in the temporary bank is lost for that trial, and the next trial begins. The BART task consisted of 60 balloon trials, one third of which were low, medium or high payoff value (0.5 cents, 1.0 cents, and 5.0 cents per pump). These three levels of monetary reward were included to provide a step function of reward (White et al., 2007, 2008).

SI Table 1

Personality and Change in Task-induced Exuberance and Anger over Time

Personality Measures	Induced Exuberance Day 2 *r (p)*	Induced Anger Day 2 *r (p)*	Change in Induced Exuberance (d1 to d2) *r (p)*	Change in Induced Anger (d1 to d2) *r (p)*
Trait Agency				
Social Potency	.20 (.11)	-.05 (.38)	-.15 (.18)	**-.32 (.023)***
**Discriminant Measures (Non-agentic)**				
Trait Anxiety				
Stress Reaction	-.05 (.78)	.15 (.35)	**.25 (.07)$^{+}$**	.07 (.33)
Trait Impulsivity				
Control	.02 (.89)	-.17 (.31)	.12 (.24)	-.15 (.18)
Trait Affiliation				
Social Closeness	-.02 (.91)	.09 (.57)	**-.32 (.025)***	-.01 (.47)
Trait Fear/Cautious Timidity				
Harm Avoidance	.03 (.86)	.12 (.46)	.04 (.40)	.04 (.40)
Trait Immersive Emotion				
Absorption	.09 (.60)	-.14 (.38)	.22 (.09)	**-.35 (.015)***
Trait Interpersonal Aggression				
Aggression	.07 (.66)	.11 (.52)	.21 (.10)	-.09 (.29)

Note. Columns 1 and 2 indicate personality findings with induced exuberance and anger on day 2. In contrast to day 1, task-induced exuberance and anger on day 2 were unrelated to scores on any personality measure. Columns 3 and 4 indicate change in agentic responses to the task from day 1 (d1) to day 2 (d2). Change in induced exuberance and anger by day 2 was systematically related to personality traits that predicted original agentic reactions to the task when it was first experienced, i.e. on day 1 (findings indicated in **bold**; details in Table 3). Individuals with low trait-levels of SP, SC and SR had lower agentic reactions to the task at first exposure (per Table 3); findings in columns 3 and 4 indicate these individuals were responsible for the bulk of the change in induced exuberance and anger responses to the task by day 2. Predictable variation in agentic emotion to the task on day 2 was thus reduced on day 2, minimizing associations with induced emotion on day 2 due to a ceiling effect in PA and NA responses. *Induced Exuberance Day 2* =post-task PA minus pre-task PA on day 2. *Induced Anger Day 2* =post-task NA minus pre-task NA on day 2. *Change in Induced Exuberance d1 to d2* = Task-induced PA on day 2 minus task-induced PA on day 1. *Change in Induced Anger d1 to d2* = Task-induced NA on day 2 minus task-induced NA on day 1 (calculations in methods). Task = i-BART. Personality measures on the Multidimensional Personality Questionnaire Brief Form (MPQ-BF). *N*=39, Study 1.

SI Figure 1

Tests of Convergent Validity

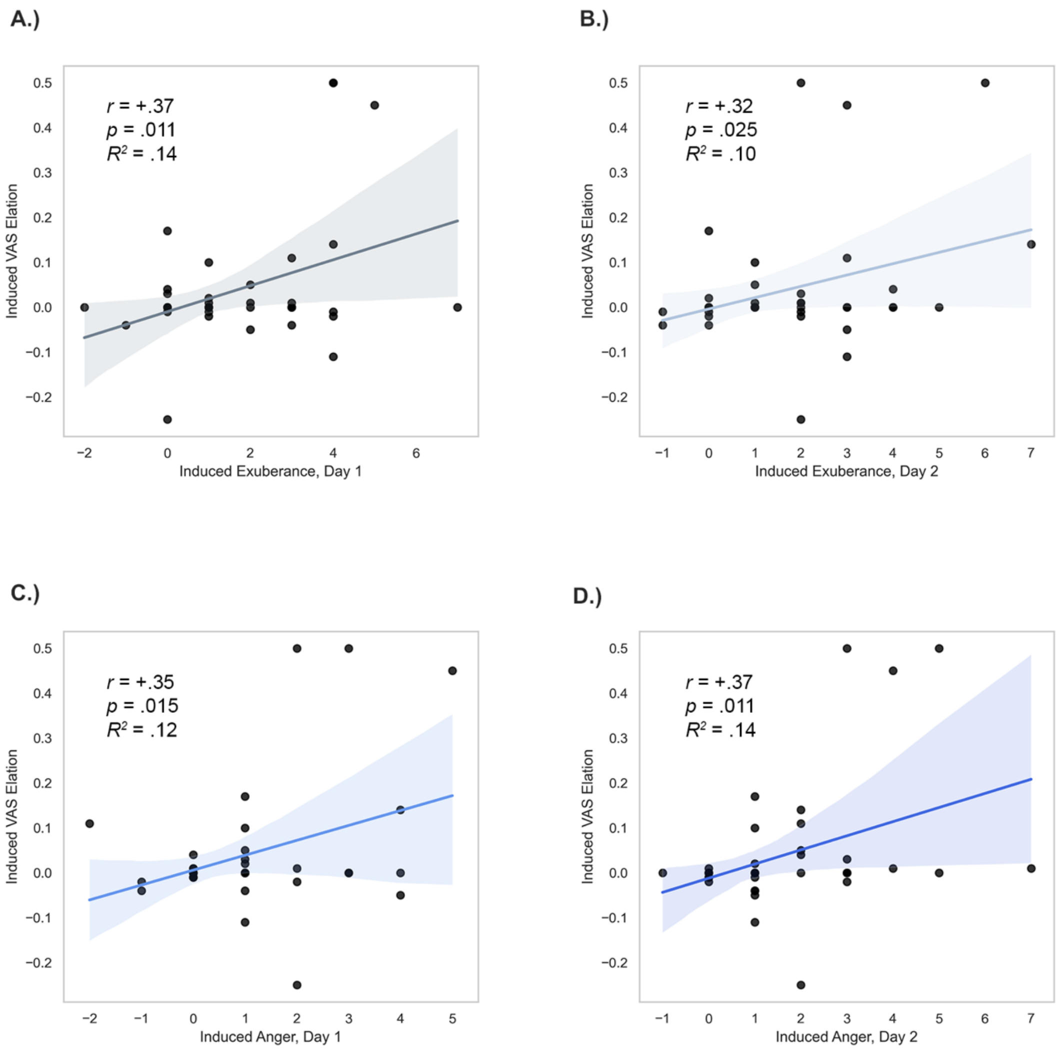

Note.
A. Day1 task-induced exuberance and task-induced VAS elation, r = +.37, p = .011, R^2 = .14.
B. Day2 task-induced exuberance and task-induced VAS elation, r = +.32, p = .025, R^2 = .10.
A. Day1 task-induced anger and task-induced VAS elation, r = +.35, p = .015, R^2 = .12.
B. Day2 task-induced anger and task-induced VAS elation, r = +.37, p = .011, R^2 = .14.
These effects were medium in size. *Induced Exuberance* = Post-task PA minus pre-task PA. *Induced Anger* = Post-task NA minus pre-task NA. *Induced VAS Elation* = Post-task VAS Elation minus pre-task VAS Elation (details in convergent validity; methods). Task = i-BART. N=39, Study 1.

SI Table 2

Tests of Discriminant Validity

	Induced Exuberance		Induced Anger	
**Discriminant States**	**Day 1** ***r (p)***	**Day 2** ***r (p)***	**Day 1** ***r (p)***	**Day 2** ***r (p)***
Subjective Arousal				
POMS Arousal[a]	-.10 (.55)	.18 (.29)	-.02 (.89)	.12 (.48)
Physiological Arousal				
Diastolic BP[b]	.20 (.22)	.27 (.10)	.14 (.38)	-.04 (.80)
Systolic BP[b]	.16 (.32)	.16 (.33)	.12 (.48)	.15 (.38)
HR[b]	-.07 (.66)	.25 (.13)	-.02 (.91)	-.06 (.73)
Subjective Anxiety				
POMS Anxiety[a]	.19 (.27)	-.01 (.94)	-.09 (.60)	.26 (.12)
VAS Anxiety[a]	.15 (.36)	.12 (.47)	.13 (.43)	.06 (.73)

Note. Task-induced exuberance and anger were unrelated to task-induced change in subjective arousal, subjective anxiety and cardiovascular activity. No relationships reached significance ($p < .05$) and no relationships were significant after Bonferroni correction for multiple comparisons (adjusted alpha=.008). *Induced Exuberance* = post-task PA minus pre-task PA. *Induced Anger* = post-task NA minus pre-task NA. Task = i-BART. POMS = Profile of Mood States. BP = blood pressure. HR = heart rate. VAS = Visual Analogue Scale. Task-induced change in subjective arousal, physiological arousal, and subjective anxiety provide information on discriminant validity (calculations in methods). [a]N=38, [b]N=39, Study 1.

SI Figure 2

ANOVA Results, Drug by Stakes Interaction in Right Nucleus Accumbens

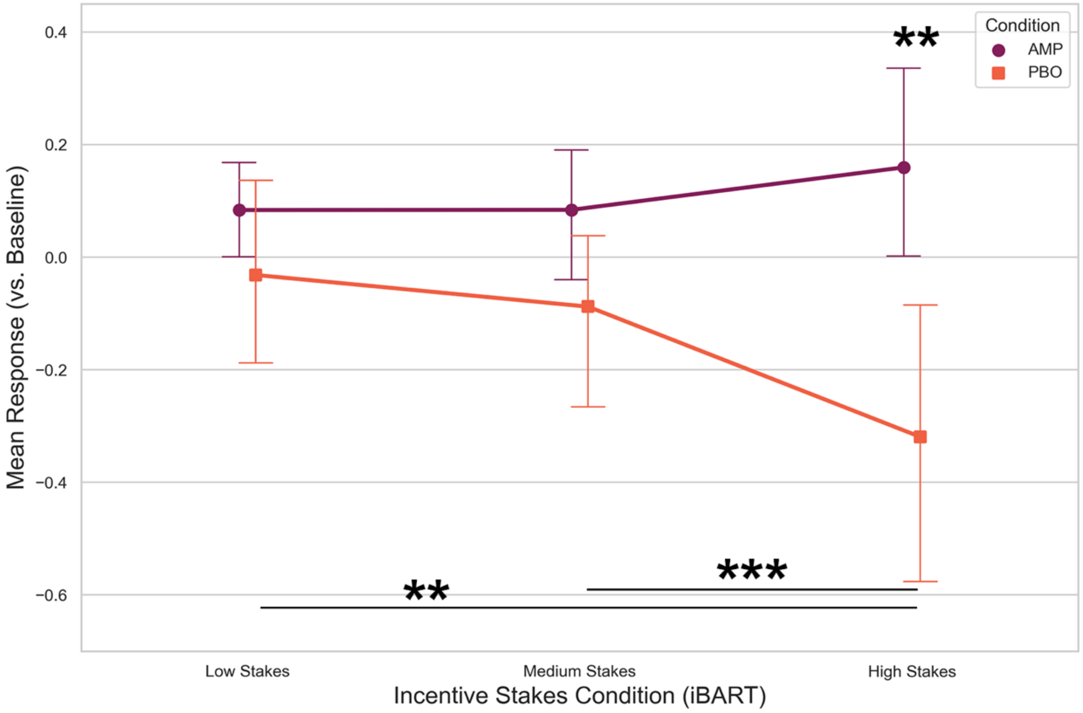

Note. There was a significant drug x stakes interaction ($F(2,18)=5.14$, $p=.017$) on activity in the right nucleus accumbens ROI. The drug x stakes interaction qualifies activation to stakes in the region. Red line indicates PBO. Blue line indicates AMP. During PBO, activation was significantly lower during HS blocks than MS blocks ($t(9)=3.45$, $p=.0035$) and LS blocks ($t(9)=2.77$, $p=.011$). AMP reversed this pattern, with increased BOLD activation overall (significant main effect of drug, $F(1,9)=5.93$, $p=.038$); and greatest activity during HS blocks. There was higher BOLD activation to HS under AMP than PBO ($t(9) = 2.73$, $p=.012$). AMP effects on BOLD activity rose as a function of incentive stakes on the fMRI task ($d=.51$, .70, 1.32 for AMP effect on LS, MS, and HS activity). Task = i-BART. Baseline is response during the sensorimotor control blocks, in which participants actively finger-pressed to a $0 condition of the task (thereby providing a conservative control for visual and motor aspects of the task). ***$p<.005$ (PBO MS vs PBO HS: $p = .0035$). **$p=.01$ (PBO LS vs PBO HS: $p = .01$; AMP HS vs. PBO HS: $p = .01$). $N=10$, Study 2.
